# Lopinavir Derivative as Potent P‑gp Inhibitor Enables Delivery through HPMA Copolymer Conjugates and Overcoming Tumor Chemoresistance to Conventional Cytostatic Drugs

**DOI:** 10.1021/acs.biomac.5c02097

**Published:** 2026-01-19

**Authors:** Daniil Starenko, Libor Kostka, Katerina Behalova, Lenka Kotrchova, Vladimir Subr, Jirina Kovarova, Radka Roubalova, Milada Sirova, Tomas Etrych, Marek Kovar

**Affiliations:** † 86863 Institute of Microbiology of the Czech Academy of Sciences, Videnska 1083, Prague 142 00, Czech Republic; ‡ 86879 Institute of Macromolecular Chemistry of the Czech Academy of Sciences, Heyrovskeho namesti 1888, Prague 162 00, Czech Republic

## Abstract

Tumor chemoresistance caused by P-glycoprotein (P-gp) expression in cancer cells remains a significant challenge in cancer chemotherapy. Herein, a novel P-gp-inhibiting lopinavir derivative (LD) was synthesized via esterification of protease inhibitor lopinavir with 5-methyl-4-oxohexanoic acid. LD proved to be a potent P-gp inhibitor with EC_50_ ∼ 1 μM, capable of considerable sensitization of P-gp-expressing cancer cells to conventional cytostatic drugs in vitro. The oxo functional group introduced in LD allowed its covalent linkage with the *N*-(2-hydroxypropyl)­methacrylamide copolymer carrier via a pH-sensitive hydrazone bond (P-LD). Polymer conjugation enhanced the pharmacological properties of LD in vivo, increasing its half-life in the bloodstream, protecting it from metabolic degradation, and promoting its accumulation in tumors via the enhanced permeability and retention effect. P-LD exhibited P-gp-inhibitory activity and sensitized cells to polymer-bound cytostatic drugs in vitro. Importantly, P-LD remarkably improved the antitumor efficacy of a polymer-bound doxorubicin in two P-gp-expressing mouse tumor models without exhibiting any systemic toxicity.

## Graphical abstract

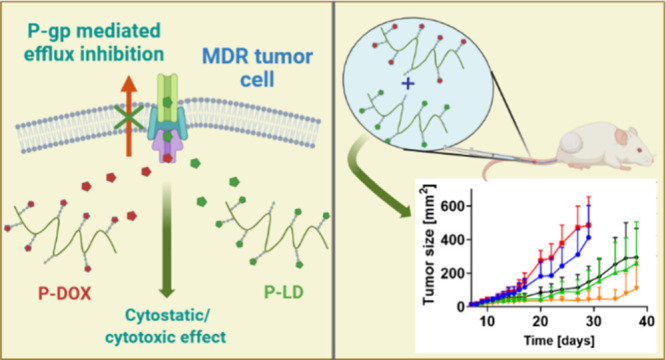

## 1 Introduction

Multidrug resistance (MDR) significantly reduces the efficacy of chemotherapy in cancer, with patients responding poorly to a wide spectrum of structurally and mechanistically unrelated anticancer drugs.^1^ Tumors may naturally exhibit MDR prior to first exposure to the chemotherapeutic agent; this phenomenon is called intrinsic MDR. It may be found in tumors derived from cells that express MDR-associated molecules under normal physiological conditions. Examples of such tissues include epithelia of the digestive system or lungs.^2–6^ Alternatively, tumors may develop MDR after several cycles of chemotherapy; such a phenomenon is called acquired MDR. It results from long-term selection pressure on tumor cells following repeated exposure to cytostatic agents.^7^ Acquired MDR has been well documented in hematological malignancies, such as acute myeloid leukemia.

One of the most common and clinically important molecular mechanisms responsible for MDR is the overexpression of P-glycoprotein (P-gp), a member of the adenosine triphosphate (ATP)–binding-cassette (ABC) transporter family. This large family of ATP-dependent membrane transporters also includes proteins such as multidrug resistance protein 1 (MRP1) and breast cancer resistant protein (BCRP), which have been reported to play a role in MDR.^8^ P-gp has a broad substrate specificity, which is determined mainly by the hydrophobicity of the transported molecules. Many anticancer compounds are hydrophobic; P-gp therefore plays a role in the efflux of a wide spectrum of unrelated drugs.^9–11^

Three generations of P-gp inhibitors have hitherto been developed. The first generation included repurposed calcium channel blockers such as verapamil; however, significant toxicity was observed at the concentrations required for achieving P-gp inhibition. The issue of toxicity was addressed with second-generation drugs, such as dexverapamil and gallopamil, via structural alterations of the first-generation drugs. The third-generation drugs include a set of P-gp-specific inhibitors (zosuquidar and elacridar), which were developed based on a structure–activity relationship-based approach. Short hydrophobic peptides known as reversins exhibiting the capacity to alter the ATP-ase activity of P-gp were also evaluated,^12–16^ as were human immunodeficiency virus (HIV) protease inhibitors (PIs), which belong to another class of drugs. Initially developed as antiretroviral therapeutics capable of inhibiting HIV protease, PIs additionally demonstrated antitumor activity in patients with Kaposi’s sarcoma, a common HIV-associated malignancy.^17–19^ Subsequent investigations revealed the ability of several PIs to inhibit P-gp and sensitize tumor cells to the activity of cytostatic drugs.^20^ Nonetheless, none of the above-mentioned inhibitors are currently used in a clinical setting. High toxicity resulting from off-target P-gp inhibition in healthy tissues upon coadministration of these inhibitors with conventional cytostatic drugs was the most significant problem identified in clinical trials.

Drug delivery systems (DDSs) are promising tools for lowering the adverse effects of P-gp inhibitors, while simultaneously improving their solubility and pharmacokinetics. Different DDSs, such as liposomes, inorganic carriers (including gold and silver nanoparticles), and polymeric carriers, have been described to date.^21–23^ Multiple studies have confirmed the efficacy of DDSs in delivering P-gp inhibitors and cytostatic drugs.^24–28^ Polymeric DDSs have been shown to be highly versatile, with the capacity to facilitate controlled drug release when the drug is covalently bound to the polymeric carrier via an enzymatically cleavable, reducible, or pH-sensitive bond.^29,30^ Moreover, DDSs have been shown to facilitate increased drug accumulation in solid tumors via the enhanced permeability and retention (EPR) effect, described as the retention of macromolecules of >40 kDa molecular weight in solid tumors because of their leaky vasculature and poor lymphatic drainage.^31,32^ Thus, controlled drug release and EPR effect may increase the therapeutic-to-adverse effects ratio due to the selective accumulation of the polymer–drug conjugate and subsequent release of the drug to its pharmacologically active form in the tumor tissue, while attenuating the exposure of healthy tissues to the active form of the drug. A water-soluble biocompatible polymer carrier based on *N*-(2-hydroxypropyl)­methacrylamide (HPMA) is among the most promising DDSs. The biocompatibility and nontoxicity of HPMA copolymers have been demonstrated in numerous studies.^33,34^ We have previously shown that P-RD, a HPMA copolymer conjugate bearing RD (ritonavir [Rit] derivatized with 5-methyl-4-oxohexanoic acid [MeOHe] to enable covalent linkage with the carrier via a pH-sensitive hydrazone bond), can be employed for efficiently overcoming P-gp-mediated MDR and inhibiting the proteasome pathway as well as signal transducer and activator of transcription 3 (STAT3) signaling in cancer cells.^30,35^ Rit was the first PI approved for the treatment of HIV infection; however, several other PIs are presently available. Given this background, we aimed to develop a more potent P-gp inhibitor by derivatizing available PIs to enable covalent linkage with the HPMA copolymer via a hydrazone bond, which requires an oxo functional group.

In the current study, derivatives of PIs (PIDs) were obtained via esterification of Rit and five other PIs with MeOHe to introduce the oxo functional group. The derivative of lopinavir (Lop), hereinafter termed LD, demonstrated the highest P-gp inhibitory activity among all of the PIDs evaluated herein, exceeding that of RD; the HPMA-based polymer conjugate of LD (P-LD) was therefore prepared. In vitro experiments revealed that P-LD markedly sensitized cancer cells with both acquired and intrinsic MDR to the cytostatic and cytotoxic activities of two conventional cytostatic drugs bound to the HPMA copolymer. Finally, combination therapy with P-LD and an HPMA copolymer conjugate bearing doxorubicin resulted in antitumor activity superior to monotherapy and demonstrated negligible toxicity in mouse models of cancer with acquired and intrinsic MDR bearing progressively growing P388/MDR- and CT26-derived tumors, respectively.

## 2 Materials and Methods

### 2.1 Synthesis of PIDs and HPMA-Copolymer-Based Conjugates

#### 2.1.1 Materials

Methacrylic anhydride; 1-amino-propan-2-ol; 2,2′-azobis­(2-methylbutyronitrile) (AIBN); di*tert*-butyl dicarbonate (BOC); BOC-hydrazide; 4-(dimethylamino)­pyridine (DMAP); *N,N-*dimethylacetamide (DMAc); *N*-ethyl-*N*′-(3-(dimethylamino)­propyl)­carbodiimide hydrochloride (EDC); 2,4,6-trinitrobenzenesulfonic acid (TNBSA); dimethyl sulfate; ethanethiol; carbon disulfide; dichlormethane (DCM); sodium hydride (60% dispersion in mineral oil); and silica gel 60 were purchased from Sigma-Aldrich (Czech Republic). 2,2′-Azobis­(4-methoxy-2,4-dimethylvaleronitrile) (V-70) was purchased from Wako Fujifilm. Ritonavir (TCI, Japan), Lopinavir, Saquinavir (Across Organics, USA), Indinavir sulfate, and Nelfinavir mesylate (Cayman Chemical, USA), Atazanavir sulfate (Molekula, UK), Docetaxel (TCI, Japan), and Doxorubicin (Maiji Seiko, Japan)

#### 2.1.2 Monomer and Chain Transfer Agent (CTA)

The monomers *N*-(2-hydroxypropyl)­methacrylamide (HPMA) and *N*-(*tert*-butoxycarbonyl)-*N*′-(6-(methacryloylamino)­hexanoyl)­hydrazine (Ma-Ah-NHNH-BOC) were synthesized as described previously.^30^ Purity of the monomers was confirmed by ^1^H NMR.

The CTA, *S*-2-cyano-2-propyl-*S*-ethyl trithiocarbonate (ethylTTc-A), was synthesized as described by Ishitake et al.^36^ CTA was characterized by ^1^H NMR.

#### 2.1.3 PIDs and Docetaxel Derivative

LD was synthesized by esterifying Lop with 5-methyl-4-oxohexanoic acid (MeOHe), catalyzed by DMAP. Lop (1.1 g, 1.75 mmol), DMAP, and MeOHe (0.303 g, 2.10 mmol) were dissolved in DCM (15 mL), and then EDC (0.7 g, 3.50 mmol) was added. The mixture was stirred at room temperature for 4 h and purified using a PrepChrom C700 FLASH chromatograph (Büchi, Switzerland) on Chromolith prep RP-18e 100–25 mm (mobile phase water/acetonitrile (ACN) with gradient 0–100% ACN, Merck, Germany) with UV detection at 220 nm. The yield was 0.750 g (70.3%). Molar masses of derivatives present in the article were calculated in ACD/ChemSketch (ACD/Laboratories, Canada). Following the same procedure, docetaxel (DTX) was derivatized, gaining DTX derivative (DTXD). DTXD was prepared by esterifying levulinic acid via carbodiimide coupling. DTX (0.6 g, 0.74 mmol), DMAP, and levulinic acid (103 mg, 0.89 mmol) were dissolved in DCM (10 mL), and then EDC (341 mg, 1.78 mmol) was added. The reaction was stirred at room temperature for 18 h in the dark. The final derivative was isolated using a PrepChrom C700 FLASH chromatograph (Büchi, Switzerland) on Chromolith prep RP-18e 100–25 mm (mobile phase ACN with gradient 0–100% ACN) with UV detection at 220 nm. The yield was 0.405 g (60.1%).

#### 2.1.4 Polymer Precursors and Conjugates

Polymer precursor poly­(HPMA-*co*-Ma-Ah-NHNH-BOC) was prepared by reversible addition–fragmentation chain-transfer (RAFT) copolymerization of HPMA and Ma-Ah-NHNH-BOC using V-70 as an initiator and ethylTTc-A as a CTA in molar ratios of monomer:CTA:initiator 500:2:1. The molar ratio of HPMA to Ma-Ah-NHNH-BOC in the reaction mixture was 94:6. HPMA (10.0 g, 69.83 mmol), Ma-Ah-NHNH-BOC (1.397 g, 4.46 mmol), 61.03 mg CTA (0.297 mmol) and 45.83 mg (0.149 mmol) V-70 was dissolved in mixture of DMAc/*t*-BuOH (1/9 (v/v)). Oxygen from the reaction mixture was removed by argon flow, and the polymerization ampule was sealed and incubated at 30 °C for 72 h. Polymer precursor was isolated and purified from residual monomers via precipitation into aceton/diethyl ether (3/1 (v/v)) and reprecipitated from methanol into aceton/diethyl ether (1/1 (v/v)). Trithiocarbonate polymer end groups were removed by reaction with AIBN in DMAc according to the method described by Perrier et al.^37^ The final polymer precursor with free hydrazide groups was obtained by BOC deprotection in water at 100 °C for 40 min.^38^

Polymeric conjugates bearing LD, doxorubicin (DOX), and DTXD were synthesized analogously to P-RD, as described earlier.^35^ Briefly, the polymer precursor with free hydrazide groups (2400 mg) was dissolved in dry methanol (20.0 mL), and LD (200 mg) was added, followed by the addition of acetic acid (1.6 mL). The reaction mixture was stirred for 20 h at room temperature. The reaction mixture was precipitated into a mixture of ethyl acetate/DCM and reprecipitated into pure ethyl acetate. The conjugate yield was 2300 mg, and the LD content determined via HPLC calibration was 6.6 wt %.

### 2.2 Characterization Method Used for Prepared Compounds

#### 2.2.1 Nuclear Magnetic Resonance (NMR)

^1^H NMR spectra were collected with the same parameters on a Bruker Avance II 400 MHz instrument (Bruker, USA). The width of the 90° pulse was 18 μs, the relaxation delay was 10 s, and the acquisition time was 2.18 and 16 scans. Chemical shifts were calibrated on the d6-DMSO signal (δ = 2.5 ppm). All samples were filled into 5 mm NMR tubes. The spectra were processed via the software TopSpin 4.5.0 (Bruker, USA).

#### 2.2.2 High-Performance Liquid Chromatography (HPLC)

CTA and drug derivatives were analyzed using a HPLC Shimadzu system equipped with an SPDM20A photodiode array detector (Shimadzu, Japan) with a reverse-phase column Chromolith HighResolution RP-18e, 150 × 4.6 mm (Merck, Germany). Gradient elution was performed with 5–95% acetonitrile containing 0.1% of trifluoroacetic acid (TFA) for 7 min at a flow rate of 4.0 mL/min.

#### 2.2.3 Size Exclusion Chromatography (SEC)

The weight-average molecular weight (*M*_w_), number-average molecular weight (*M*_n_), dispersity (*Đ*), and hydrodynamic volume (*R*_h_) of the synthesized polymer compounds were measured using SEC on an HPLC Shimadzu system equipped with four detectors: a SPDM20A photodiode array detector (Shimadzu, Japan), a differential refractometer (OptilabrEX), a multiangle light-scattering (DAWN HELLEOS II) detector, and a viscometric detector (ViscoStar III, all from Wyatt Technology Co., USA). Additionally, the HPLC system was equipped with a DGU-20A5R degasser, an LC-20AD pump (both from Shimadzu, Japan), and columns Superose-6-Increase 10/300 GL (Cytiva, USA) and a CBM-20A controlling unit (Shimadzu, Japan). The mobile phase used for sample elution was 0.05 M phosphate buffer +0.15 M NaCl at pH 7.4. The flow rate was 0.5 mL/min. Data analysis was performed using Astra 8.1.2 software (Wyatt Technology Co., USA).

### 2.3 Antibodies

Primary antibodies against STAT3 (anti-STAT3/124H6, Cell Signaling Technologies, USA), phosphorylated STAT3 (anti-STAT3/Y705, Abcam, UK), β-actin (Santa Cruz Biotechnologies, USA); secondary antimouse-IgG conjugated with horseradish peroxidase, and antirabbit-IgG conjugated with horseradish peroxidase (Cell Signaling Technology, USA) were used in the experiments.

### 2.4 Cell Lines

The CT26 mouse colon adenocarcinoma cell line (catalog no. CRL-2638, RRID: CVCL_7256) was purchased from the American Type Culture Collection (ATCC, Manassas, VA, USA) and cultivated in RPMI-1640 medium (Sigma-Aldrich, Czech Republic) supplemented with heat activated fetal bovine serum (10%), 100 U/mL of penicillin-streptomycin solution, 1 mM sodium pyruvate, 4.5 g/L of glucose, and 10 mM HEPES. The SCC7 mouse head and neck squamous cell carcinoma cell line was kindly gifted by Dr. Deanne M.R. Lathers from The Medical University of South Carolina (Charleston, USA) and cultivated in RPMI-1640 medium supplemented with heat-activated fetal bovine serum (10%) and 100 U/mL of penicillin-streptomycin solution. P388 mouse monocytic leukemia and P388/MDR mouse monocytic leukemia with multidrug resistance cell lines were kindly gifted by Professor I. Lefkovits from Basel Institute for Immunology (Switzerland) and cultivated in RPMI-1640 medium supplemented with heat-activated fetal bovine serum (10%), 100 U/mL of penicillin-streptomycin solution, 1 mM sodium pyruvate, and nonessential amino acids (1%). DOX (750 ng/ml) was added to the cultivation medium of the P388/MDR cell line to maintain its chemoresistant phenotype. Cell cultures were kept at conventional cultivation conditions (37 °C, 5% CO_2_ atmosphere) to about 80–90% confluence and subcultured up to four times before thawing a new vial of frozen cells. We routinely screen our cell lines for mycoplasma using the MycoAlert Mycoplasma Detection Kit (Lonza, Switzerland).

### 2.5 Animal Models

DBA/2 (H-2^d^) inbred strain mice were obtained from Charles River’s breeding facility (Charles River, Sulzfeld, Germany). BALB/c (H-2^d^) inbred mice and Rag2^–/–^ immune-deficient mice on the BALB/c genetic background were obtained from the animal facility of the Institute of Microbiology of the Czech Academy of Sciences, v.v.i. Mice were provided with water and food *ad libitum*. Mice used for experiments were 9–15 weeks old and in 20–25 g body weight range. All animal work was conducted according to institutional guidelines for the care and use of laboratory animals and strictly followed the protocol (AVCR 2755/2021 SOV II) approved by the Institutional Animal Care and Use Committee of the Academy of Sciences of the Czech Republic, as well as conducted in compliance with local and European guidelines.

### 2.6 Real-Time Polymerase Chain Reaction (RT-qPCR)

Relative normalized expression of ABC transporters in selected cell lines was determined on RNA isolated from the cells using real-time RT-qPCR

#### 2.6.1 Primers for RT-qPCR

Antisense and sense primers of *ABCB1, ABCC1, ABCG2, CASC3, ACTB*, and *HPRT* mouse genes for real-time PCR were ordered from Generi Biotech (Czech Republic).

#### 2.6.2 RNA Isolation

RNA was isolated from P388/MDR, P388, CT26, and SCC7 cell lines incubated for 48 h. 2–4 × 10^6^ cells were used for each cell line isolation procedure. Cells were incubated with TRIzol nucleic acid isolation solution (Ambion, USA) for 5 min at room temperature. Nucleic acids were extracted from the lysates by mixing them with isopropanol. Samples were washed twice with 75% ethanol and resuspended in 20 μL of ultraclean PCR water. Concentrations of RNA isolated from cells were counted using a NanoDrop 2000 spectrophotometer (Thermo Fischer Scientific, USA) at 260 nm after 15 min of incubation at 60 °C.

#### 2.6.3 Reverse Transcription

2 μg of isolated RNA were used for the reverse transcription procedure. Before transcription, samples were treated with DNase (TURBO DNA-free kit, Thermo Fischer Scientific, USA) and incubated for 30 min at 37 °C to remove contaminating genome DNA. Oligoseoxythimidine deoxyribonucleotide mixture (Genri Biotech, Czech Republic) and ultraclean PCR water were added to samples, followed by 5 min incubation at 65 °C. Reaction mix containing reverse transcription buffer solution, 10 mM DTT, RNase OUT ribonuclease inhibitor, and SuperScript IV reverse transcriptase (Thermo Fischer Scientific, USA) was used for reverse transcription. The mixture was incubated in a PCR-cycler (10 min/50 °C; 10 min/80 °C). Product purity was evaluated by agarose gel electrophoresis using transcriptase-free samples as controls.

#### 2.6.4 RT-qPCR

FrameStar PCR plates (4titude, Germany) were used to perform a real-time PCR reaction. Reaction mix was prepared by combining sense and antisense primers for *ABCB1*, *ABCC1*, and *ABCG2* genes, gb SG PCR master mix (Generi Biotech, Czech Republic), ultraclean PCR water, and previously prepared cDNA from cells (20× diluted). Measurement of amplification curves was performed on CFX 96 Touch RT-qPCR device (BioRad, USA) with cycling program 10 min/95 °C; 10 s/94 °C, 25 s/58 °C, 35 s/72 °C (40 cycles); melting curve detection for 1 min/54 °C and gradient 54–95 °C. Evaluation of the results was conducted in the CFX Manager program (BioRad, USA). Expression levels were normalized to *ActB*, *Hprt*, and *CasC3* genes and presented as a means of relative normalized expression from two wells ± SD. The experiment was conducted twice with similar results.

### 2.7 Calcein Efflux Assay

P388/MDR or CT26 cells (1.5–2 × 10^5^/well) were seeded into a 96-well flat-bottom plate (Nunc, Denmark) in 100 μL of cultivation medium. Titrated concentrations of PIs, PIDs, and P-LD diluted in medium were added to cells to reach a final volume of 200 μL. Cells were incubated for 30 min (for PIs and PIDs) or for 3, 6, 9, and 16 h (for P-LD). Cells with medium added instead of the samples were used as a negative control, and cells with medium containing 10 μM cyclosporine A were used as a positive control. Acetoxymethyl ester of calcein (calcein; Thermo Fischer Scientific, USA) was added to each well (0.2 μM), followed by 30 min of incubation in the dark. Cells were washed 3x with flow cytometry buffer (2% FTS, 2 mM EDTA in PBS). Flow cytometry measurement was conducted in TPP U-well plates (TPP, Switzerland) on an LSR II flow cytometer (BD, USA). At least 50,000 live cells were acquired per well, and Hoechst 33258 (0.1 μg/mL; Thermo Fisher Scientific, USA) was used to determine dead cells. Data were evaluated using FlowJo software (Tree Star, Inc., Ashland, OR, USA; FlowJo, RRID:SCR_008520). Results are shown as mean fluorescence intensity of triplicate ±SD, and each experiment was conducted at least twice with similar results.

### 2.8 LogP Calculation

Calculation of the octanol/water partition coefficient logarithm (LogP) was performed via free access online software XlogP3 Online^39^ using chemical structures of PIs and PIDs made in ACD/ChemSketch (ACD/Laboratories, Canada).

### 2.9 Western Blotting

CT26 cells were incubated (15 h) with Rit, RD, Lop, or LD and stimulated for the last 1 h of incubation with mIL-6 (100 ng/mL). Cells were washed two times with ice-cold PBS and lysed using mammalian protein extraction reagent (M-PER, 78501, Thermo Fisher Scientific, USA) with an added halt protease and phosphatase inhibitor cocktail (78447, Thermo Fisher Scientific, USA) for 1 h on ice. The protein content was quantified via bicinchoninic acid (23227, Thermo Fisher Scientific, USA). Each sample (25 μg of protein) was separated by SDS-PAGE and transferred to nitrocellulose membranes via a semidry blotting system (Bio-Rad, USA). 5% nonfat dry milk in TBST buffer (50 mM Tris, 150 mM NaCl, and 0.05% Tween 20) was used to block nitrocellulose membranes (1 h, room temperature), followed by incubation with primary antibody (4 °C, overnight). After the incubation, membranes were thoroughly washed with TBST and incubated (1 h, room temperature) with a secondary either antirabbit or antimouse Ig antibody (1/10,000) conjugated with horseradish peroxidase. Membranes were washed again with TBST and incubated in Restore Stripping Buffer (21059, Thermo Fisher Scientific). Protein loading was verified with anti-β-Actin antibody (1/1000). G-Box (TECAN, Switzerland) was used to detect chemiluminescence after the membranes were incubated with SuperSignalTM West Femto Maximum Sensitivity Substrate (34095, Thermo Fisher Scientific).

### 2.10 Enzyme-Linked Immunosorbent Assay (ELISA)

Preparation of cell lysates was performed in the same way as that for Western blotting analysis (2.9). The experiment was performed using a PathScan Total Stat3 Sandwich ELISA kit (Cell Signaling Technology, USA) and a PathScan P-Stat3 (Tyr705) Sandwich ELISA kit (Cell Signaling Technology, USA) according to the manufacturer’s instructions. Absorbance was measured on an Infinite 200 microplate reader (Tecan, Switzerland) at a 450 nm wavelength.

### 2.11 [^3^H]-Thymidine Incorporation Assay

[^3^H]-Thymidine incorporation assay was used to evaluate the cytostatic effect of selected PIDs and P-LD and their combinations with polymeric conjugates bearing conventional cytostatic drugs. Cell suspension of P388/MDR (5 × 10^3^ cells/well), CT26 (1 × 10^4^ cells/well), or SCC7 (1 × 10^4^ cells/well) were seeded into a 96-well bottom tissue culture plate with flat bottom (Nunc, Denmark) before titrated concentrations of tested compounds were added to reach a final volume of 250 μL. Cells incubated with medium only were used as a negative control. Cells were incubated for 72 h; 50 μL of [^3^H]-Thymidine (4 μCi/mL; 25× diluted; PerkinElmer, USA) was added to each well for the last 6 h of incubation. The plates were harvested on a membrane (1450-421 Printed Filtermat, PerkinElmer, USA) using Harvester 96 (TOMTEC, Germany). Scintillation of the harvested sample DNA was measured on Microbeta 2450 Microplate counter (PerkinElmer, USA) using a plastic melt-on scintillator sheet (PerkinElmer, USA). The activity of controls was >20,000 cpm/well in all experiments. Results are shown as representative titration curves assembled from means ± SD from tetraplicates of each cytostatic drug concentration, with IC_50_ shown in parentheses in the legend. The same applies to IC_50_ plots with IC_50_ values ± SD. Each experiment was conducted at least twice with similar results.

### 2.12 Annexin V Assay

Apoptosis induction by combinations of LD (or P-LD) and cytostatic drugs or their polymeric conjugates was evaluated by an annexin V assay detecting outer membrane acetyl serine. Cell suspensions of P388/MDR (5 × 10^5^ cells/well) and CT26 (1.5 × 10^5^ cells/well) cells in 2 mL of cultivation media were treated with a titrated concentration of the tested compounds to reach a final volume of 2.5 mL in the well of a 6-well flat-bottom tissue culture plate (Thermo Fisher Scientific, USA). Cells incubated with medium only were used as a negative control. Cells were incubated for 48 h. After incubation, cells were filtered through 30 μm filters (Sysmex, Germany) and washed twice with annexin binding buffer (10 mM HEPES, 8.2 g/L NaCl, and 280 mg/L CaCl_2_ in distilled H_2_O) in 96-well conical bottom plates (Thermo Fisher Scientific, USA). After washing, cells were resuspended in 20 μL of 50x-diluted Annexin V-Dyomics 647 (Exbio, Czech Republic) dye and incubated on ice for 30 min. Next, Hoechst 33258 (0.1 μg/mL) and the annexin binding buffer were added to reach a volume of 100 μL. 50,000 cells for each experimental condition were analyzed using the LSRII flow cytometer and FlowJo software.

### 2.13 Caspase-3 Assay

Assay was performed using an EnzCheck Caspase-3 Assay Kit (Thermo Fisher Scientific, USA). Cells suspensions of P388/MDR (1.25 × 10^6^ cells/well) or CT26 (1 × 10^6^ cells/dish) cells were treated with titrated concentration of tested samples to reach a final volume of 2.5 mL in 6-well flat bottom tissue culture plate for P388/MDR and 10 mL in a Petri dish (Thermo Fisher Scientific, USA) for CT26 cells. Cells incubated with medium only were used as a negative control. Cells were incubated for 48 h. After incubation, approximately 3 × 10^6^ cells were used for each sample assessment. Cells were washed with PBS, resuspended in 230 μL of cell lysis buffer, and lysed on ice for 45 min. Lysates were then centrifuged (10,000 × *g*, 5 min), and supernatants (50 μL) were transferred into the well of a 96-well flat-bottom plate and mixed with 50 μL of reaction buffer containing substrate Z-DEVD-AMC. A 50 μL portion of lysis buffer was used as a blank, and a standard dilution series containing free AMC was prepared for the generation of a calibration curve. The plate was incubated for 30 min at room temperature. Fluorescence measurement was performed by an Infinite 200 microplate reader (Tecan, Switzerland) using a 342 nm wavelength for excitation and a 441 nm wavelength for emission detection. Results are presented as the mean ± SD of the released AMC amount from triplicate. Each experiment was conducted twice with similar results.

### 2.14 Evaluation of Toxicity and Antitumor Activity In Vivo

Toxicity and antitumor activity in vivo were evaluated in several mouse models. Experiments evaluating P-LD antitumor effect were conducted in BALB/c mice bearing progressively growing CT26 tumors. 150 or 200 mg/kg of P-LD was applied i.p. on days 8, 10, 12, 14, and 16. Rag2^–/–^ male mice and DBA/2 female mice with progressively growing P388/MDR tumors were used for the determination of the toxicity and antitumor activity of the P-DOX and P-LD combination. Rag2^–/–^ mice were injected with 1 × 10^6^ P388/MDR cells; P-DOX (30, 40, or 50 mg/kg i.v) and P-LD (120, 160, or 200 mg/kg, i.p) were injected on day 6. DBA/2 mice were injected with 2.5 × 10^5^ P388/MDR cells s.c. into the left flank; P-DOX (25 mg/kg, i.v.) and P-LD (100 mg/kg, i.p.) were injected on days 6, 9, and 12. BALB/c female mice bearing progressively growing CT26 tumors were also used to evaluate the toxicity and antitumor activity of P-DOX and P-LD combination. Mice were injected with 2 × 10^5^ CT26 cells s.c. into the left flank; P-DOX (30 mg/kg, i.v.) and P-LD (120 mg/kg, i.p.) were injected on days 7, 10, and 13. Doses in all cases are given as equivalents of drugs bound to a polymeric carrier. Control groups with no treatment or only monotherapy treatment were included in combination therapy experiments. Body weight of experimental animals, tumor growth, and survival were monitored.

### 2.15 Statistical Analysis

Statistical analysis was performed using an unpaired two-tailed Student’s *t*-test for group comparison in in vitro experiments and in vivo tumor growth analysis; Mantle–Cox log-rank test was used for survival analysis. The statistical evaluations were performed using GraphPad Prism (GraphPad Software, USA). Differences with *P* ≤ 0.05, *P* ≤ 0.01, and *P* ≤ 0.001 were considered statistically significant.

## 3 Results and Discussion

### 3.1 Synthesis and Characterization of PI Derivatives

The drugs Lop, Indinavir (Ind), Atazanavir (Atz), Saquinavir (Saq), Nelfinavir (Nel), and Rit were selected herein. All of these drugs lack a suitable chemical group for direct conjugation with polymer carriers. We preferred to introduce the hydrazone bond, a pH-sensitive spacer, between the selected drugs and a polymeric precursor, as this has been previously reported as a highly useful pH-sensitive tool for such types of molecules.^28,35,40^ Consequently, the synthesis of PIDs was initiated via esterification of the active ingredients with MeOHe to introduce the oxo functional group. Overall, six derivatives were synthesized, and their structures were confirmed by using NMR spectroscopy. The NMR spectra of Rit-MeOHe (RD, 847.1 g/mol), Lop-MeOHe (LD, 755.0 g/mol), Ind-MeOHe (ID, 740.0 g/mol), Saq-MeOHe (SqD, 797.0 g/mol), Atz-MeOHe (AD, 831.0 g/mol), and Nel-MeOHe (ND, 694.0 g/mol) are shown in Figures S1–S6. All of the synthesized PIDs are depicted in Figure 1.

**Figure 1:**
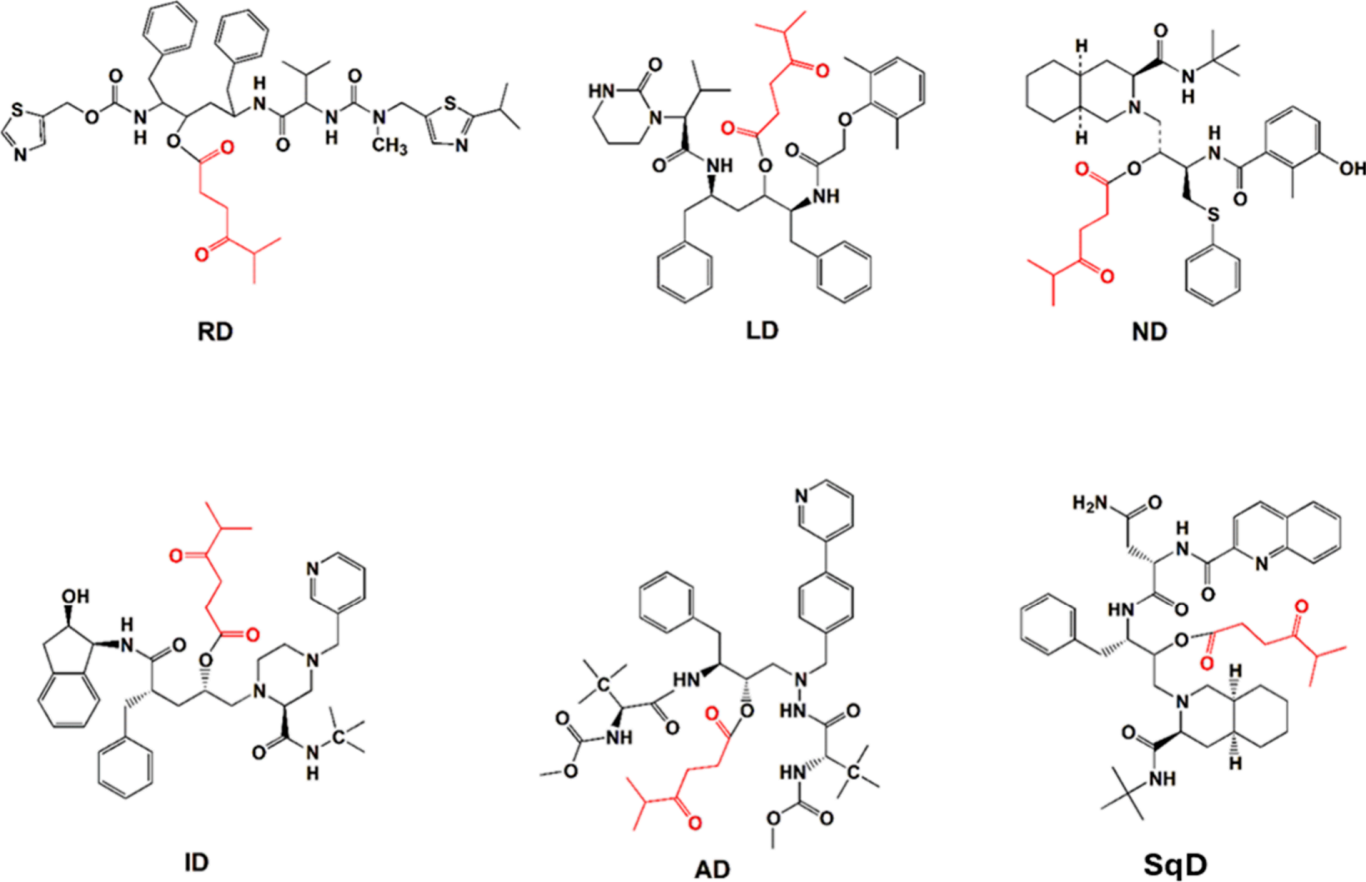
Structures of derivatives of the selected drugs synthesized herein and their corresponding abbreviations.

### 3.2 LD Is a Highly Potent P-gp Inhibitor That Also Inhibits the STAT3 Signaling Pathway

Six PIs approved for clinical use by the United States Food and Drug Administration, including Rit, Lop, Ind, Atz, Nel, and Saq, were modified via esterification with MeOHe to introduce the oxo functional group. The P-gp-inhibitory potential of these PIDs was evaluated under in vitro conditions to enable the selection of the best candidate for the synthesis of the polymer–drug conjugate. Prior to this evaluation, the best cell lines capable of serving as model systems for induced and intrinsic MDR in various types of cancer were selected herein. The cell line P388/MDR (mouse monocytic leukemia with high P-gp expression induced by prolonged DOX exposure) was selected as the model system for induced MDR. Similarly, cell lines CT26 (mouse colon carcinoma) and SCC7 (mouse squamous head and neck carcinoma) were chosen as model systems for intrinsic MDR. RT-qPCR was employed for assessing the expression of three genes encoding ABC transporters (*Abcb1*, *Abcc1*, and *Abcg2* encoding P-gp, MRP1, and BCRP, respectively), which can play a role in the development of MDR in cancer cells. The cell line P388 was used as the negative control. P-gp was found to be highly expressed in the P388/MDR cells. Moreover, the expression of P-gp was slightly elevated in CT26 and SCC7 cells compared to that in P388 cells, indicating that P-gp is expressed to a certain extent in these tumor cells under physiological conditions (Figure S7). Interestingly, high MRP1 expression was observed in the SCC7 cells. These three cell lines were, therefore, used for subsequent experiments.

The P-gp-inhibitory potential of the above-mentioned PIs and their derivatives was evaluated using calcein efflux assay and flow cytometry analysis in P388/MDR cells; cyclosporin A (CsA) was employed as the positive control (Figures 2A–F and S8A,B). The P-gp inhibitory activities of PIDs were higher than those of the PIs, with Nel and ND being the only exceptions. This can be explained, at least in part, by the fact that MeOHe is an aliphatic molecule; derivatization with MeOHe is, therefore, expected to increase the overall hydrophobicity of the PIs. This premise was validated by computing the octanol–water partition coefficient (LogP) of these compounds (Figure S8C). Notably, derivatization with MeOHe increased the P-gp-inhibitory activity of Ind from nil to moderate. This observation can be explained by the fact that the relative increase in the hydrophobicity of Ind after derivatization is higher than that of the other PIs (Figure S8D). LD was the most potent P-gp inhibitor among all of the compounds evaluated herein, with ∼50% P-gp inhibition achieved at a lower concentration of LD (1 μM) than that of CsA (10 μM). LD thus has considerably higher potency than the previously described RD.^28,30,35^ Another advantage of LD over RD is that Lop, in contrast to Rit, is not an inhibitor of cytochrome P450 3A4 (CYP3A4), which metabolizes a broad spectrum of cytostatic drugs.^41–43^ In turn, this means that combination therapy involving RD and some cytostatic drugs, such as DTX, can lead to markedly increased toxicity. Indeed, significantly increased toxicity was observed upon the coadministration of P-RD and the HPMA copolymer conjugate bearing DTXD (unpublished results). Such an outcome is unlikely in the case of LD. An analysis of P-gp inhibition by Rit, Lop, and Ind as well as their derivatives was also conducted in the cell lines CT26 (Figure S8E–G) and SCC7 (data not shown). P-gp inhibition in these cells was not as prominent as that in P388/MDR cells because of lower levels of P-gp expression; nevertheless, the strong P-gp-inhibitory activity of the LD was confirmed.

**Figure 2:**
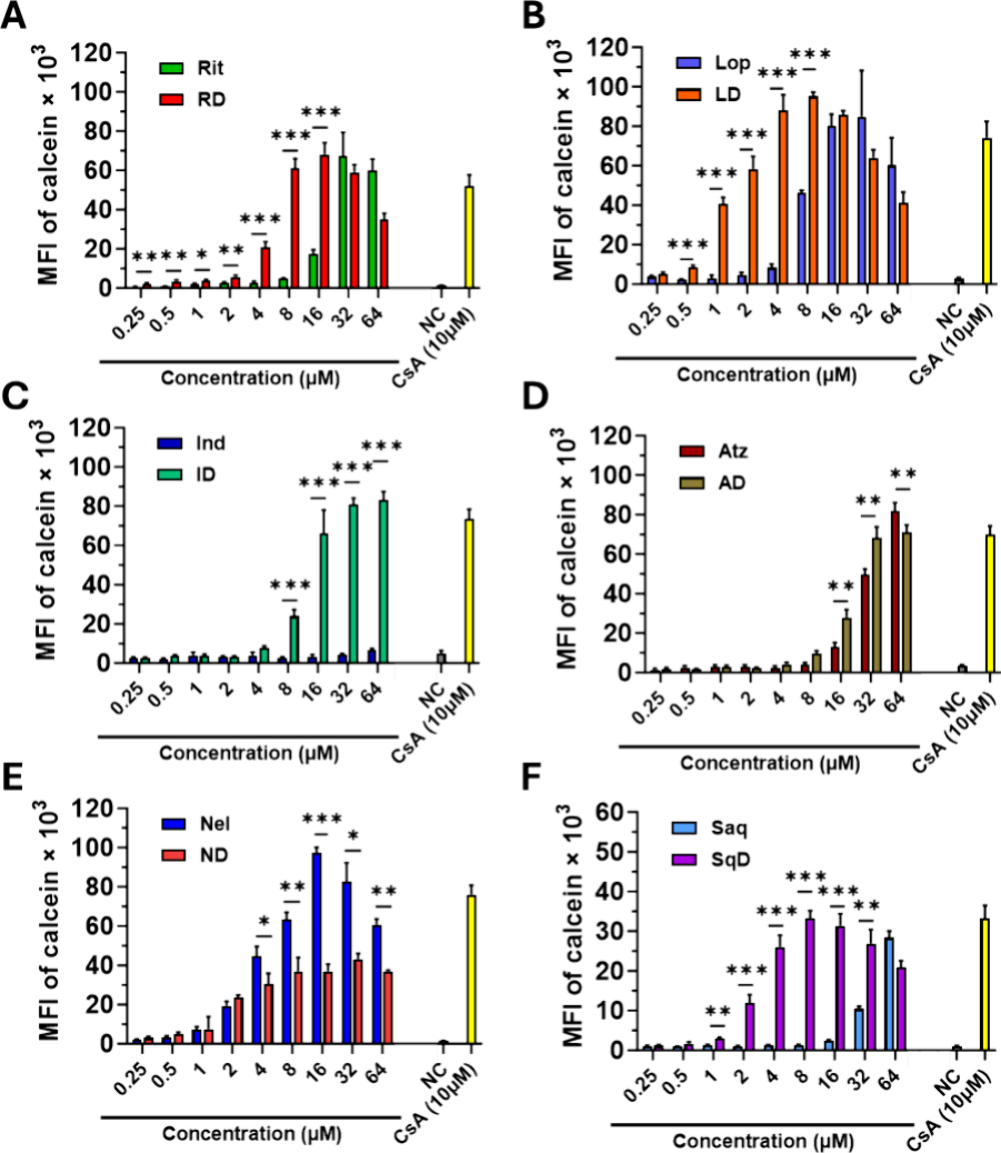
Derivatization of the selected PIs with MeOHe to enable covalent linkage with the HPMA copolymer carrier via pH-sensitive hydrazone bond alters their P-gp-inhibitory activity. P-gp-inhibitory activity of selected PIs and their derivatives in P388/MDR cells following a 30 min incubation (A–F). Titrated concentrations of Rit or RD (A), Lop and LD (B), Ind and ID (C), Atz and AD (D), Nel and ND (E), and Saq and SqD (F) were determined using calcein efflux assay and flow cytometry analysis. P388/MDR cells incubated with 10 μM CsA or only incubation medium were employed as positive control and negative control (NC), respectively. Each bar represents the mean ± standard deviation (SD) of the measured values of mean fluorescence intensity (MFI) of calcein from triplicate samples. Experiments were conducted at least twice and yielded similar results. Statistically significant differences between compared compounds evaluated via unpaired two-tailed Student’s *t*-test are indicated by *, **, and ***, denoting *P* ≤ 0.05, *P* ≤ 0.01, and *P* ≤ 0.001, respectively.

In addition to P-gp inhibition, the potential of Rit, RD, Lop, and LD to inhibit STAT3 phosphorylation was evaluated using Western blot analysis and ELISA (Figure S9A,B). Significant inhibition of STAT3 phosphorylation was observed upon treatment with all the evaluated compounds, with LD demonstrating the strongest effect. Furthermore, LD not only inhibited the phosphorylation of STAT3 but also decreased the overall expression of the protein in treated cells. The inhibition of STAT3 phosphorylation by PIs such as Rit, Nel, and Lop has been extensively reported; however, none of these compounds decreased the total levels of STAT3.^44–46^ Taken together, these results suggest that LD is a potent P-gp inhibitor that additionally inhibits STAT3 signaling, a pathway that is possibly associated with the development of chemoresistance in cancer cells by increasing Bcl-2 expression and overall survival.^47,48^ Thus, the P-gp and STAT3 inhibitor LD was selected for incorporation into the HPMA copolymer conjugate for subsequent in vivo studies.

### 3.3 Synthesis and Characterization of Polymer–Drug Conjugates

Linear polymer conjugates based on HPMA that incorporated LD, DTXD, or DOX were synthesized in multiple steps. This process was initiated with the RAFT copolymerization technique, which ensured precise control over polymer size.^49,50^ The sizes of all synthesized compounds were adjusted to promote prolonged circulation in the body while allowing for the elimination of the polymer carrier by the kidneys.^51^ Immediately after polymerization, polymer precursors with ω-TTc end groups were obtained. These end groups were later removed via a reaction with an excess of AIBN in DMAc, a method adapted from Perrier et al.^37^ Next, BOC protecting groups were removed from the hydrazide groups using TFA. The resulting polymer precursor with hydrazide groups was used for synthesizing polymer-based therapeutics containing LD, DTXD, or DOX covalently bound via a pH-sensitive hydrazone bond. The characteristics of the synthesized polymer conjugates are listed in Table 1.

**Table 1:** Characteristics of Polymer Precursor and Polymer Conjugates with LD, DTXD, and DOX

precursor/conjugates	abbreviation	molar mass^a^ [g/mol]	*Đ* ^a^	content of derivative^b^ [wt %]	hydrodynamic radius^c^ [nm]
polymer with hydrazides	P	35,400	1.09		4.3
polymer-MeOHe-Lop	P-LD	38,800	1.1	5.7	4.3
polymer-DTXD	P-DTXD	37,200	1.1	6.7	4.3
polymer-DOX	P-DOX	30,600	1.1	7.2	3.5

a Weight-average molar mass and dispersity (*Đ*) of polymers were determined using SEC-MALS-dRI analysis.

b Content of drug derivatives in the polymer conjugates was determined using high-performance liquid chromatography from DAD detector, as described in Section 2.2.2.

c Determined via online differential viscometer during SEC analysis.

### 3.4 P-LD Possesses Significant Potential to Inhibit P-gp under In Vitro Conditions

The P-gp-inhibitory potential of P-LD was compared to that of free LD using P388/MDR cells and incubation periods of 45 min and 16 h for LD and P-LD, respectively (Figure 3A). The P-gp-inhibitory activity of LD was higher than that of P-LD at lower concentrations, but their activities were comparable at higher concentrations; this effect may be explained by the gradual release of the drug from the polymer carrier. To ascertain the association between the incubation period and the P-gp-inhibitory activity of P-LD, the extent of P-gp inhibition by P-LD was evaluated after different incubation periods (Figure 3B). The P-gp-inhibitory activity of P-LD was found to be clearly incubation time-dependent within the incubation period evaluated herein (3–16 h), demonstrating the gradual release of LD from the HPMA copolymer carrier. Moreover, P-LD proved to be a more potent P-gp inhibitor than P-RD based on analogous experiments with P-RD conducted in a previous study from our group. Only 50% of P-gp inhibition relative to that of the positive control was attained therein with 8 μM P-RD after 24 h of incubation, whereas 75% was achieved herein with the same concentration of P-LD after 16 h of incubation.^35^ These results prove that P-LD is a potent inhibitor of P-gp via the gradual release of LD.

**Figure 3:**
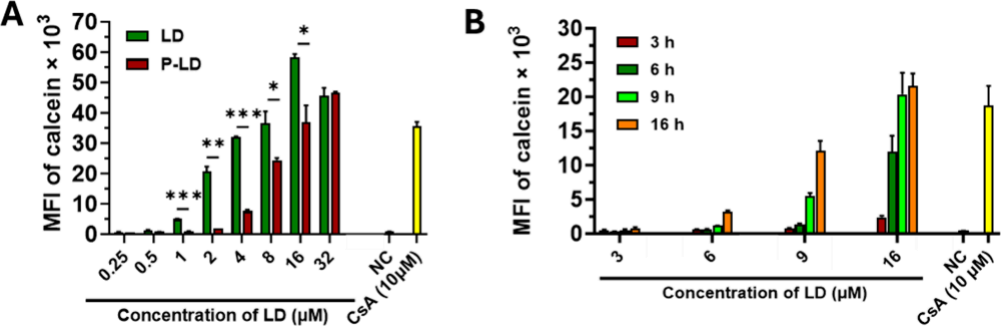
P-gp-inhibitory activity of P-LD is conserved after conjugation with the HPMA polymer via a pH-sensitive hydrazone bond, which enables gradual release from the carrier. P-gp-inhibitory activity of LD and P-LD in P388/MDR cells after 45 min and 16 h of incubation, respectively (A). Kinetics of P-gp inhibition following incubation of cells with titrated concentrations of P-LD for 3, 6, 9, and 16 h (B). P388/MDR cells incubated with 10 μM CsA and only incubation medium were employed as positive control and NC, respectively. Concentrations of P-LD are shown as equivalents of free LD. Each bar represents the mean ± standard deviation (SD) of the measured values of mean fluorescence intensity (MFI) of calcein from triplicate samples. Experiments were conducted at least twice and yielded similar results. Statistically significant differences between compared compounds evaluated via unpaired two-tailed Student’s *t*-test are indicated by *, **, and ***, denoting *P* ≤ 0.05, *P* ≤ 0.01, and *P* ≤ 0.001, respectively.

The release profiles of LD from the HPMA polymer conjugate in solutions of differing pH values that mimic blood (pH 7.4) and the lysosomal compartment of tumor cells (pH 5.0) are shown in Figure S10. The results reveal that >85% of LD is released within 5 h at pH 5.0, while only 18% is released over 24 h at pH 7.4. LD release is therefore significantly higher at pH 5.0, confirming the acidic pH-facilitated hydrolysis of the polymer conjugate and its stability under conditions modeling the bloodstream. Moreover, degradation of the ester bonds present in the LD structure was not observed in either of the buffers with differing pH over the time frame evaluated herein.

LD contains a ketone group capable of reacting with endogenous nucleophiles, especially thiols such as glutathione and the cysteine residues of proteins, resulting in the formation of hemithioacetal adducts. This reaction can modify the original molecule, potentially changing its pharmacological activity, stability, or biodistribution. Such adduct formation can also influence the interpretation of biological efficacy, as the active species may not solely be a free LD but also a conjugated form. Further investigations, such as mass spectrometry analysis using cell cultures, can help determine the presence and importance of such derivatives. However, such experiments are beyond the scope of this study and can be addressed separately.

### 3.5 LD and P-LD Significantly Sensitize Tumor Cells Expressing P-gp to the Cytostatic Effect of Clinically Relevant Anticancer Drugs and Their Polymer-Bound Conjugates

Before the potential of LD and P-LD to sensitize P-gp-expressing cells to cytostatic drugs was evaluated, their inherent cytostatic activity was first assessed to identify concentrations that exhibited limited effects on proliferation in the selected cancer cell lines (Table S1). The cytostatic activity of LD was approximately 1.5–2.5 times higher than that of Lop, while that of P-LD was up to 2-fold lower than that of LD in the cancer cell lines employed herein. Furthermore, Lop and LD demonstrated higher cytostatic activities than those of Rit and RD, respectively. Based on these results, LD and P-LD were used at concentrations of 0.5–4 μM (for the polymer conjugate, corresponding equivalent concentrations of LD were used) for sensitizing cells to the cytostatic activity of DOX or DTX and their corresponding HPMA copolymer-bound counterparts. Higher concentrations (2–16 μM equivalents of LD) of P-LD were used in some experiments to achieve more significant sensitization. Thus, P388/MDR, CT26, and SCC7 cells were incubated with titrated concentrations of free cytostatic drugs and several constant concentrations of LD (Figure 4A).

**Figure 4:**
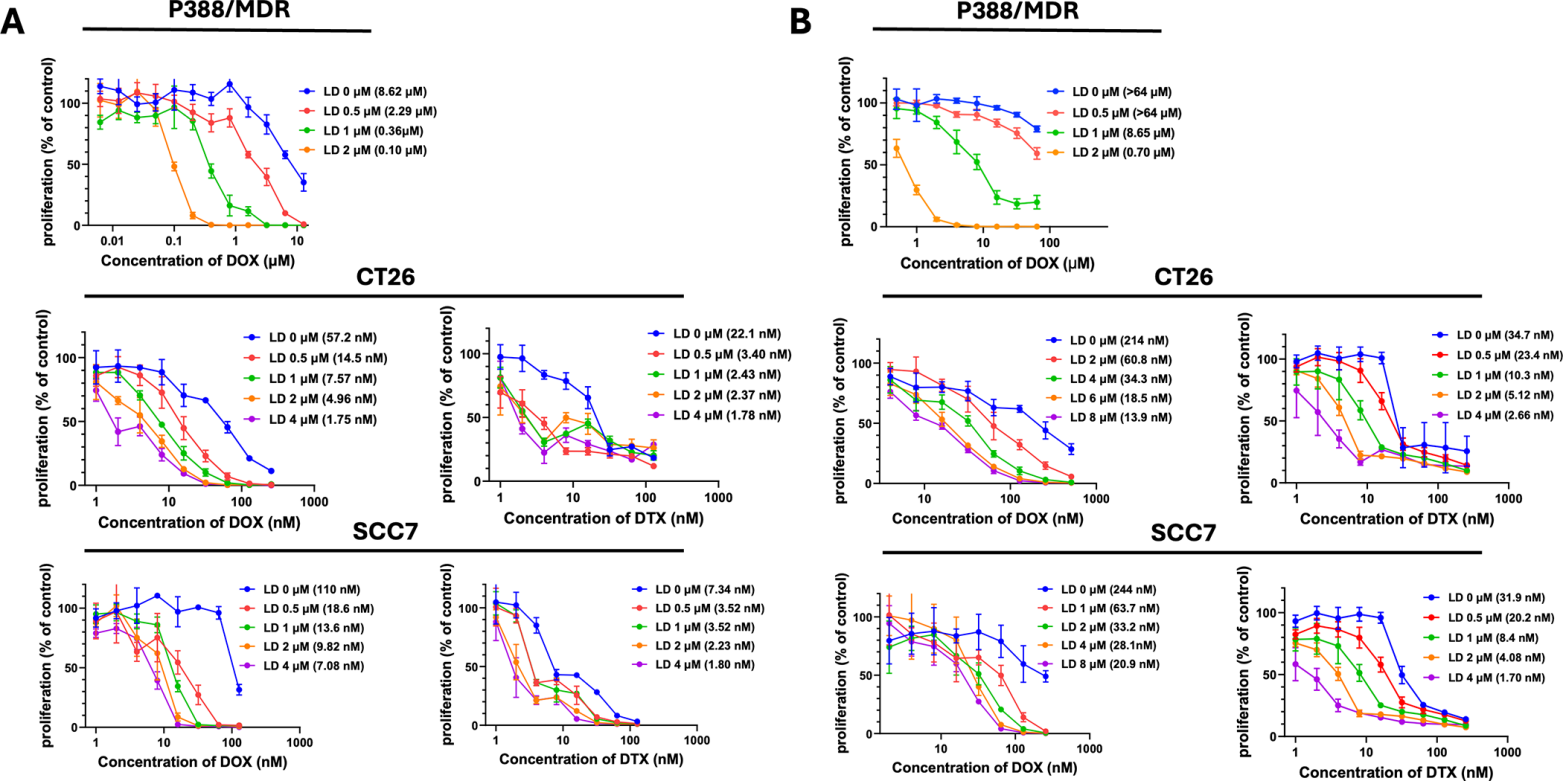
LD and P-LD cause potent sensitization of P-gp-expressing cancer cells to the cytostatic activity of free and HPMA copolymer–bound conventional cytostatic drugs, respectively. Sensitization of P388/MDR, CT26, and SCC7 cells to the cytostatic activity of DOX or DTX in the presence of LD at various constant concentrations (A) or to that of P-DOX or P-DTXD at various constant concentrations of P-LD (B) after 72 h of incubation. [^3^H]-thymidine incorporation assay was employed herein. Concentrations shown in experiments with polymer conjugates represent free drug equivalents. Proliferation of cells exposed to the test drugs relative to those exposed to the same concentration of only LD or P-LD have been plotted. IC_50_ values for each cytostatic drug in the absence or presence of LD or P-LD are presented in brackets. Each data point represents the mean ± SD of the tetraplicate samples. Each experiment was conducted at least twice, and similar results were obtained.

LD was highly potent in sensitizing P388/MDR cells to the cytostatic activity of DOX, resulting in approximately 86-fold lower IC_50_ value of the drug compared to that in cells incubated with DOX alone (Figure 4A upper panel). Moreover, a highly significant sensitization of P388/MDR cells to the cytostatic activity of P-DOX was obtained in the presence of P-LD, although the effects were not as potent as those achieved with the free cytostatic drug and LD (Figure 4B upper panel).

Subsequently, combinations of DOX or DTX with LD or those of their corresponding polymer conjugates were evaluated in the CT26 and SCC7 cells. LD sensitized CT26 cells more effectively to the cytostatic activity of DOX than to that of DTX, resulting in ∼32- and 12-fold reduction, respectively, in the IC_50_ values of these drugs (Figure 4A, middle panel). The sensitization of SCC7 cells was of even lower efficiency (∼16- and 4-fold, respectively; Figure 4A bottom panel). The lower efficiency of LD-mediated sensitization of CT26 and SCC7 cells to cytostatic drugs compared with that of P388/MDR cells probably reflects the notably lower expression of P-gp in these cells relative to that in P388/MDR cells. Moreover, the difference in the extent of sensitization of the cells to DOX and DTX may be attributable to the fact that DTX is a poorer substrate for P-gp than DOX, which has been previously experimentally validated.^52^ The combinations of P-DOX or P-DTXD with P-LD followed the same trend, but with somewhat lower overall extent of sensitization than in the case of free drugs (Figure 4B middle and bottom panels), a finding similar to that described above with P388/MDR cells. The only exception was the combination of P-DTXD and P-LD in SCC7 cells (Figure 4B bottom panel), which, interestingly, resulted in a greater extent of sensitization than the combination of DTX and LD (Figure 4A bottom panel). The IC_50_ values were separately plotted and are presented in Figure S11.

The chemosensitizing effects of the previously described P-gp inhibitor RD^28,35^ and ID developed herein were also evaluated in combination with DOX in P388/MDR (both derivatives) and CT26 cells (RD only). The chemosensitizing effects of RD and ID were weaker than those of LD, further confirming that LD was the most potent P-gp inhibitor among the selected compounds (Figure S12).

Taken together, these results reveal that LD and P-LD are capable of potently chemosensitizing cells to the cytostatic effects of conventional anticancer drugs and their HPMA copolymer-bound conjugates. This effect is more prominent in the P388/MDR cell line with very high levels of P-gp expression, but can also be observed in the CT26 and SCC7 cell lines used herein as model systems for intrinsic MDR. Another evident trend in almost all the experiments pertains to the lower cytostatic effects of the HPMA copolymer-bound drugs and slightly lower chemosensitizing effects of P-LD compared to their free drug counterparts under in vitro conditions, which is attributable to the gradual release of the drugs from the polymer carrier. However, this characteristic of polymer-bound drugs is not expected to be a disadvantage under in vivo conditions, as polymer-based carriers can prolong the half-life of drugs in circulation and afford protection from drug metabolism and accumulation in tumors due to the EPR effect.

### 3.6 LD and P-LD Significantly Augment Apoptosis Induced by Conventional Cytostatic Drugs and Their Polymer Conjugates, Respectively, in P-gp-Expressing Cancer Cells

The potential of LD to augment DOX- and DTX-induced apoptosis and that of P-LD to augment P-DOX- and P-DTXD-induced apoptosis were subsequently evaluated in P388/MDR and CT26 cells. The extent of apoptosis was determined by Annexin V/Hoechst staining, followed by flow cytometry analysis and measurements of caspase-3 activity in cell lysates. The concentrations of cytostatic drugs, their polymer conjugates, LD, and P-LD were carefully selected to allow for limited induction of apoptosis in these cell lines.

LD considerably enhanced DOX-induced apoptosis in P388/MDR cells at a concentration of 1 μM, decreasing the live cell percentage from 85 to 68% in combination-treated cells. An increase in LD concentration to 4 μM resulted in nearly complete induction of apoptosis by DOX (Figure 5A). Comparable results were obtained with the combination of P-DOX and P-LD (Figure 5G), albeit at higher concentrations, as previously observed in vitro proliferation studies.

**Figure 5:**
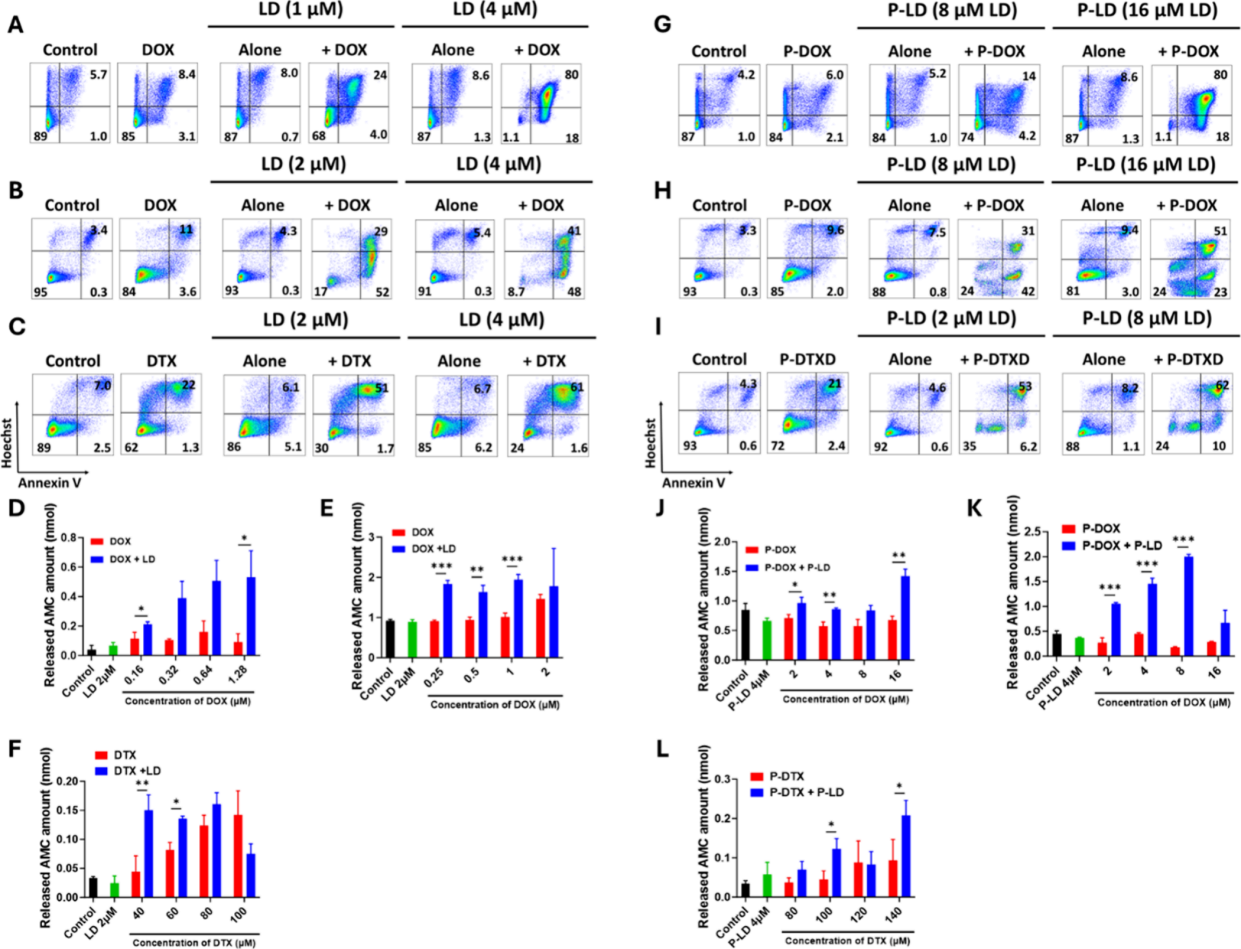
LD and P-LD considerably potentiate the induction of apoptosis in cancer cells expressing P-gp by conventional cytostatic drugs and their polymer-bound counterparts, respectively. Induction of apoptosis in P388/MDR (A, G) and CT26 (B, C, H, and I) cells was determined by annexin V–Dy647/Hoechst 33258 staining followed by flow cytometry analysis. The cells were incubated with 10 or 1 μM DOX (A and B, respectively) or 120 μM DTX (C) alone or in combination with the indicated concentrations of LD for 48 h. The cells were incubated with 80 or 8 μM (G and H, respectively) of P-DOX or 150 μM P-DTXD (I) alone or in combination with the indicated concentrations of P-LD for 48 h. Untreated cells (control) and cells treated with the corresponding concentrations of LD or P-LD alone were included as controls. Representative dot plots from one of at least two independent experiments, each with triplicate samples, which yielded similar results are shown. Caspase-3 activity was determined for titrated concentrations of DOX in P388/MDR (D) and CT26 (E) cells and titrated concentrations of DTX in CT26 cells (F) alone or in combination with 2 μM LD after 48 h of incubation via a fluorescence-based assay for detecting the activity of activated caspase-3. Caspase-3 activity was determined for titrated concentrations of P-DOX in P388/MDR (J) and CT26 (K) cells and for titrated concentrations of P-DTXD in CT26 cells (L) alone or combined with 4 μM P-LD after 48 h of incubation. For the control group, the cells were incubated with only culture medium. Concentrations are represented as drug equivalents in experiments involving polymer conjugates. Each bar showing the amount of released AMC represents the mean ± SD of triplicate samples. Experiments were conducted at least twice and yielded similar results. Statistically significant differences between compared compounds evaluated via unpaired two-tailed Student’s *t*-test are represented by *, **, and *** denoting *P* ≤ 0.05, *P* ≤ 0.01, and *P* ≤ 0.001, respectively.

Similar results were observed in the CT26 cell line. LD (2 μM) potently enhanced DOX-induced apoptosis in CT26 cells, reducing the fraction of live cells from 84 to 17% in combination-treated cells. At higher concentrations (4 μM), LD further reduced the viability of CT26 cells to 9% upon coincubation with concentrations of DOX with very low inherent cytotoxicity (Figure 5B). Similarly, P-LD enhanced P-DOX-induced apoptosis in CT26 cells when approximately 4- and 8-fold higher concentrations of P-LD and P-DOX, respectively, were used (Figure 5H). The potential of LD and P-LD to increase DTX- and P-DTXD-induced apoptosis in CT26 cells was also evaluated (Figure 5C, I). However, the enhancement in this scenario was not as pronounced as that obtained with DOX. This may be attributable to the very high difference in concentrations of DTX required for achieving cytostatic versus cytotoxic (apoptosis-inducing) effects, which is much higher than that required with DOX. The cytostatic activity of DTX was more than 2-fold higher than that of DOX in CT26 cells, corresponding to a lower IC_50_ value (22.1 and 57.2 nM, respectively; Figure 4A middle panel). However, apoptosis induction required >100-fold higher concentrations of DTX compared to those of DOX (120 and 1 μM, respectively; Figure 5B, C). This difference may be attributed to the different mechanisms of action of these two drugs. DOX has the capacity to intercalate into DNA and inhibit topoisomerase II; therefore, it is capable of inhibiting cell proliferation and inducing apoptosis at relatively low concentrations. By contrast, DTX disrupts microtubule dynamics and can potently inhibit proliferation at very low concentrations; however, much higher concentrations are required for the induction of apoptosis.^53,54^ In fact, previous studies have documented that effective induction of apoptosis in some cancer cell lines requires higher concentrations of DTX.^55–57^ Interestingly, the concentration of P-DTXD required for inducing apoptosis in combination with P-LD was only 1.2-fold higher than that of free DTX in combination with LD; this ratio is much lower than that observed for the DOX/P-DOX pair of free/polymer-bound drug (approximately 8-fold). This difference is attributable to the fact that HPMA-based polymer conjugates bearing DTX derivatives exhibit a higher rate of drug release than those carrying DOX, as demonstrated in previous studies.^58,59^

To further confirm the augmentation of apoptosis induced by the selected free and polymer-bound cytostatic drugs in the presence of LD and P-LD, respectively, the activity of caspase-3 was determined in lysates of P388/MDR and CT26 cells subjected to the above-mentioned treatments. The results of caspase-3 activity analysis were found to mirror those of Annexin V/Hoechst staining, followed by flow cytometry analysis. Significantly higher caspase-3 activity was found in P388/MDR (Figure 5D) and CT26 (Figure 5E, F) cells exposed to the combination of free drug (DOX or DTX) and LD than in cells exposed to the same concentration of the corresponding free drug alone. Similar results were obtained with the combination of a polymer-bound drug (P-DOX or P-DTXD) and P-LD in both P388/MDR (Figure 5J) and CT26 (Figure 5K, L) cell lines. In some experiments, reduced caspase-3 activity was observed in groups treated with the highest concentrations of cytostatic drugs (Figure 5F, K). This effect is likely due to the strong cytostatic activity of the drug combination, which compromises the condition of cells during the steps of cell lysis and determination of caspase-3 activity. Although the results were normalized to the total cell numbers in each group, this correction did not eliminate the above-mentioned inconsistency. Taken together, these results reveal that LD and its polymer-bound counterpart significantly potentiate the cytotoxic activities of the selected conventional cytostatic drugs and their polymer conjugates in P-gp-expressing cancer cells.

### 3.7 P-LD Augments the Therapeutic Efficacy of P-DOX in Mouse Tumor Models of Acquired and Intrinsic MDR Without Any Increase in Toxicity

The potentiation of the antitumor activity of polymer-bound cytostatic drug conjugates by P-LD and the toxicity of such combination treatment were subsequently evaluated in mouse models bearing progressively growing P388/MDR- or CT26-derived tumors. A preliminary evaluation indicated P-DOX as the most suitable polymer–cytostatic drug conjugate for combination therapy with P-LD since the polymer conjugate P-DTXD resulted in considerably enhanced toxicity upon coadministration with P-LD. Therefore, all subsequent experiments were carried out using the combination of P-DOX and P-LD.

The toxicity and antitumor activity of P-LD monotherapy were first evaluated in BALB/c mice bearing CT26-derived tumors. P-LD did not exert any toxic effects even at dosages as high as 200 mg/kg LD equivalents upon administration five times every second day, nor did it exhibit any antitumor effects (Figure S13A,B). A previous study from our group revealed the efficacy of monotherapy with P-RD both in this tumor model at a dosage corresponding to 60 mg/kg of RD and the B16F10 melanoma model.^35^ The structural differences between RD and LD apparently have a significant effect on their pharmacological activities, with the latter exhibiting higher efficiency of P-gp inhibition but lower efficacy as an antitumor drug compared to RD. The mechanisms underlying such a difference in vivo antitumor efficacy remain unclear and should be further investigated. The lack of antitumor activity of P-LD under in vivo conditions may be attributable to the fact that Lop (but not Rit) is rapidly metabolized by CYP3A4 in the liver. Such a scenario may be applicable for LD as well, despite some differences in the molecular structure of the compounds

A preliminary evaluation of the toxicity and antitumor activity of the combination of P-DOX and P-LD was carried out using immunodeficient Rag 2^–/–^ mice bearing progressively growing P388/MDR-derived tumors. The mice were administered a single dose of P-LD followed by a single dose of P-DOX after an hour; different doses were evaluated for both polymer conjugates. The polymer conjugate combination did not exhibit toxicity even at 50 and 200 mg/kg of DOX and LD equivalents, respectively (Figure S13C). Notably, significant growth inhibition of this highly chemoresistant tumor was achieved, especially at higher dosages (Figure S13D). The intraperitoneal (i.p.) route was selected for the administration of P-LD herein, as P-LD was poorly soluble at the required doses in the volume of saline suitable for intravenous (i.v.) administration. Administration via the i.p. route is generally less preferred than i.v. administration in preclinical studies. Therefore, a thorough literature review was conducted to ascertain the systemic availability of macromolecules following i.p. administration. Additionally, LD release kinetics from the polymer carrier was analyzed to ensure that i.p. administration allowed efficient transfer of P-LD into the bloodstream with minimal loss of LD. The macromolecules administered via the i.p. route tend to enter the bloodstream relatively rapidly via lymphatic vessels, thereby bypassing the portal vein and liver metabolism.^60–63^ In most cases, a substantial fraction of the administered compound is detectable in plasma within a few hours.^64–66^ The LD release kinetics data obtained for the polymer carrier (Figure S10) revealed that under physiological pH, only a small fraction of LD is released from the polymer conjugate, indicating that the conjugate reaches the bloodstream with minimal loss of LD content.

The antitumor activity of the combination of P-DOX and P-LD was subsequently evaluated in immunocompetent DBA/2 mice bearing progressively growing P388/MDR-derived tumors. The mice were administered P-DOX and P-LD every third day for a total of three doses. Significant toxicity was not observed during the treatment (Figure 6A). The combination of P-DOX with P-LD significantly inhibited tumor growth compared with the control treatment, while treatment with P-DOX alone did not (Figure 6B). Similarly, only the combination of P-DOX with P-LD significantly improved survival in the experimental animals (Figure 6C).

**Figure 6:**
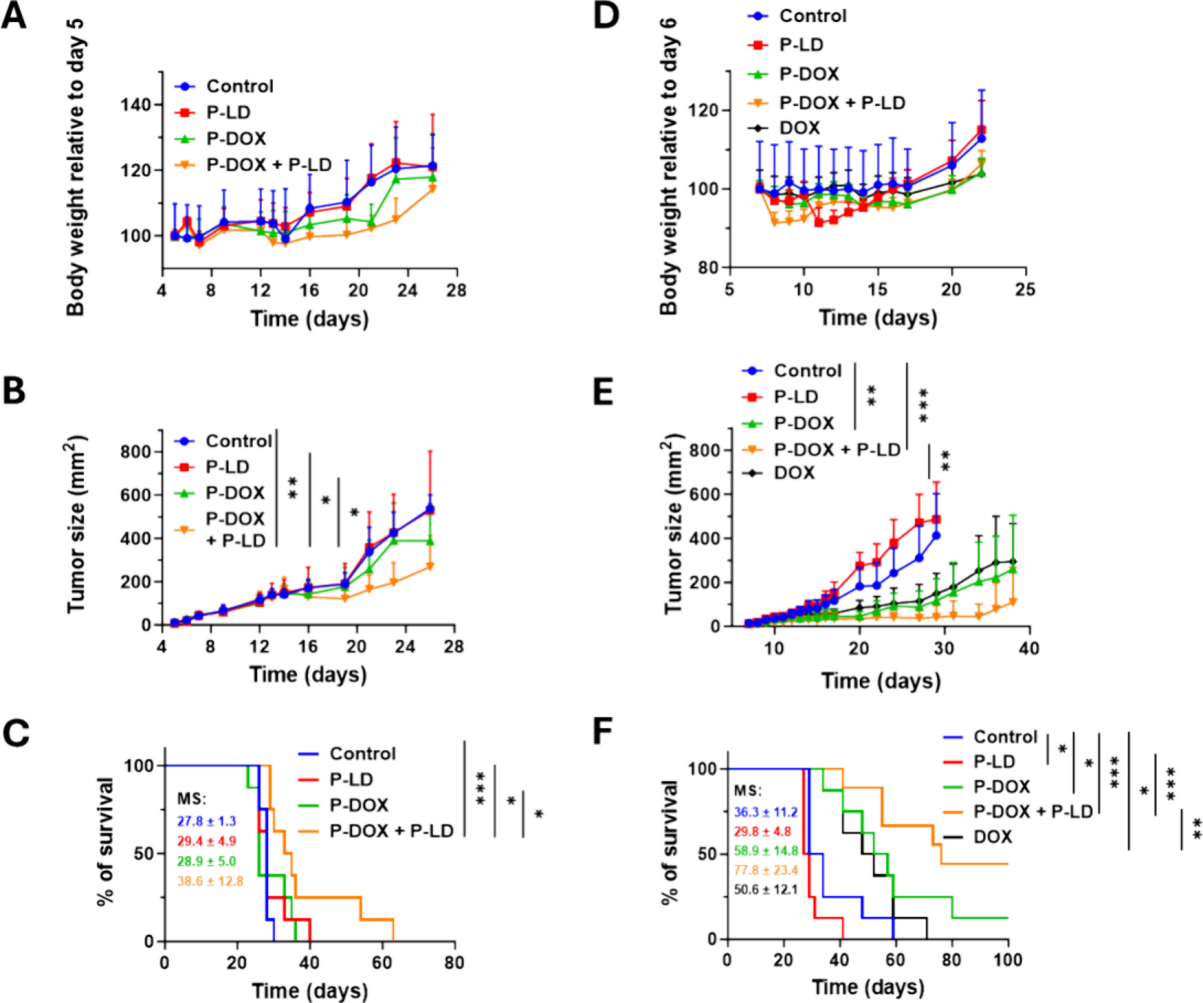
P-LD improves the antitumor efficacy of P-DOX in mouse tumor models of induced and intrinsic MDR without increasing toxicity. The combination of P-LD with P-DOX significantly inhibited tumor growth and improved survival in mice bearing P388/MDR-derived tumors. DBA/2 mice (*n* = 8) were subcutaneously (s.c.) administered 2.5 × 10^5^ P388/MDR cells on day 0. P-LD (100 mg LD/kg per dose, i.p.) and P-DOX (25 mg DOX/kg per dose, i.v.) were administered on days 6, 9, and 12. Toxicity (A), tumor growth (B), and survival of the experimental mice (C) were recorded. The combination of P-LD with P-DOX significantly inhibited tumor growth and improved survival in mice bearing CT26-derived tumors. BALB/c mice (*n* = 8; *n* = 9 for the combination treatment group) were administered 2 × 10^5^ CT26 cells on day 0. P-LD (120 mg LD/kg per dose, i.p.), P-DOX (30 mg DOX/kg per dose, i.v.), and DOX (5 mg/kg per dose, i.v.) were administered on days 7, 10, and 13. Toxicity (D), tumor growth (E), and survival of the experimental mice (F) were recorded. P-DOX was administered 1 h following the administration of P-LD in all of the experiments. Mice in the control group were injected with the same volume (250 μL) of phosphate buffered saline. Unpaired two-tailed Student’s *t*-test and Mantle–Cox log-rank test were employed for analyzing the statistical significance of the data, which has been indicated by *, **, and *** (denoting *P* ≤ 0.05, *P* ≤ 0.01, and *P* ≤ 0.001, respectively). MS denotes the mean survival in days. Experiments were done twice with comparable results.

Mice bearing CT26-derived tumors were used as the model system for intrinsic MDR for evaluating the antitumor efficacy of the combination of P-DOX and P-LD. The mice were administered P-DOX and P-LD every third day for a total of three doses, although the dosage was slightly higher than that used for the P388/MDR-derived model (30 vs 25 DOX/kg per dose for P-DOX and 120 vs 100 mg LD/kg per dose for P-LD). Toxicity was not observed even at this higher dose of P-DOX and P-LD (Figure 6D). Unlike in the P388/MDR-derived model, P-DOX alone significantly inhibited the growth of the CT26-derived tumor (Figure 6E). The combination of P-DOX with P-LD considerably potentiated the tumor inhibitory activity of P-DOX, particularly at later time points. Thus, P-LD augmented the inhibition of CT26-derived tumors by P-DOX. In turn, this prolonged the survival of mice (Figure 6F) treated with the combination (mean survival 77.8 ± 23.4 days) compared to that of mice treated with P-DOX alone (mean survival 58.9 ± 14.8 days). Moreover, the number of long-term survivors in the group treated with the combination was significantly higher than that in the group treated with P-DOX alone (four mice vs a single mouse). Notably, the results reveal that DOX is nearly as potent as P-DOX, which may contradict the claims that polymer conjugates have improved therapeutic efficacy. This discrepancy is attributable to the dosages used herein; suboptimal doses of P-DOX were employed to highlight the sensitizing effect of P-LD, whereas DOX was administered at a dose close to the maximal tolerated dose in BALB/c mice. Improvement in survival was more pronounced in the CT26-derived mouse model than in the P388/MDR-derived model. Nevertheless, the combination of polymer conjugates bearing the conventional cytostatic drug DOX and the newly developed P-gp inhibitor LD proved to be effective for the treatment of tumors with acquired as well as intrinsic MDR.

Previously published studies from our group using a combination of P-DOX and P-RD revealed a greater difference between the antitumor activities of P-DOX alone and the combination of P-DOX and P-RD.^28,35^ This is attributable to the inherent antitumor activity of P-RD, which not only chemosensitizes the tumor to the action of DOX via the inhibition of P-gp and STAT3 but also functions synergistically with DOX, considerably enhancing the antitumor activity of the combination. Such strong potentiation of DOX activity by RD was further confirmed using more advanced star-like HPMA-based DDSs to ensure better pharmacokinetic parameters and tumor accumulation of the drugs.^28^ By contrast, P-LD does not have inherent antitumor activity under in vivo conditions. The observed results are therefore attributable solely to its chemosensitization effects achieved via the inhibition of P-gp and STAT3 signaling. Thus, P-LD can be preferentially employed in scenarios where only P-gp inhibition is desired. As previously mentioned, the lack of inherent antitumor activity of P-LD under in vivo conditions may be attributed to the potential metabolism of LD by CYP3A4. As a result, P-LD may be a more suitable candidate for combinations with other anticancer drugs owing to the lower possibility of undesired toxic side effects compared with P-RD or similar conjugates based on Rit or its derivatives.

## 4 Conclusions

The selected PIs were derivatized herein to introduce an oxo functional group, enabling their covalent linkage with the HPMA copolymer carrier via a pH-sensitive hydrazone bond. LD has been identified herein as a novel potent P-gp inhibitor with superior activity compared to that of unmodified PIs and the previously described PI derivative RD. The polymer conjugate P-LD was synthesized, and its P-gp-inhibitory activity was directly confirmed under in vitro conditions. LD and P-LD significantly potentiated the cytostatic and cytotoxic activities of free and polymer-bound conventional cytostatic drugs, respectively, under in vitro conditions. P-LD was shown to be nontoxic, even at very high doses, but lacked inherent antitumor activity under in vivo conditions. P-LD considerably improved the antitumor efficacy of P-DOX without causing systemic toxicity in mouse tumor models of induced and intrinsic MDR-bearing P-gp-expressing tumors. To summarize, P-LD exhibits favorable pharmacokinetic properties and appears to be a promising, nontoxic, and effective chemosensitizer for P-gp-expressing tumors.

## Supplementary Material



## Data Availability

The authors state that the article and its Supplementary Figures contain all data which support the findings and conclusions of the study. Primary data are available from the corresponding author upon fair request.

## Notes

^1^H NMR spectra of PIDs (Figures S1–S6, PDF); Relative expression of selected ABC-transporters in cell lines used for this work determined by RT-qPCR (Figure S7, PDF); P-gp inhibitory activity relative to CsA, hydrophobicity analysis of PIDs, calcein assay in CT26 cell line (Figure S8, PDF); inhibition of STAT3 phosphorylation (Figure S9, PDF); cytostatic activity of PIs, PIDs and P-LD in selected cell lines (Table S1, PDF); Release profile of LD from HPMA copolymer at different pH levels (Figure S10, PDF); IC_50_ plots corresponding to the curves depicted on Figure 5 (Figure S11, PDF); sensitization of P-gp expressing cells to cytostatic activity of DOX via RD and ID (Figure S12, PDF); preliminary P-LD and P-LD/P-DOX combination toxicity and antitumor activity evaluation (Figure S13, PDF); and table of contents graphic for manuscript (PDF)
