# Supplementary material for: Lopinavir Derivative as Potent P‑gp Inhibitor Enables Delivery through HPMA Copolymer Conjugates and Overcoming Tumor Chemoresistance to Conventional Cytostatic Drugs
Source: Biomacromolecules. 2026 Jan 19;27(2):1510–24. doi: 10.1021/acs.biomac.5c02097 (PMC12892249; doi:10.1021/acs.biomac.5c02097)

Lopinavir derivative as potent P-gp inhibitor enables delivery through HPMA copolymer conjugate and overcoming tumor chemoresistance to conventional cytostatic drugs

Daniil Starenko[1], Libor Kostka[2], Katerina Behalova[1], Lenka Kotrchova[2], Vladimir Subr[2], Jirina Kovarova[1], Radka Roubalova[1], Milada Sirova[1], Tomas Etrych[2,], Marek Kovar[1,*]*

[1] Institute of Microbiology of the Czech Academy of Sciences, Videnska 1083, Prague, 142 00 Czech Republic

[2] Institute of Macromolecular Chemistry of the Czech Academy of Sciences, Heyrovskeho namesti 1888, Prague, 162 00 Czech Republic

[*]Corresponding authors:

Marek Kovar, Institute of Microbiology of the Czech Academy of Sciences, Videnska 1083, 14220 Prague, Czech Republic

Email: makovar@biomed.cas.cz Phone: +420 296 442 107

Tomáš Etrych, Institute of Macromolecular Chemistry of the Czech Academy of Sciences, Heyrovskeho namesti 1888, Prague, 162 00 Czech Republic

Email: etrych@imc.cas.cz, Phone: +420 296 809 231

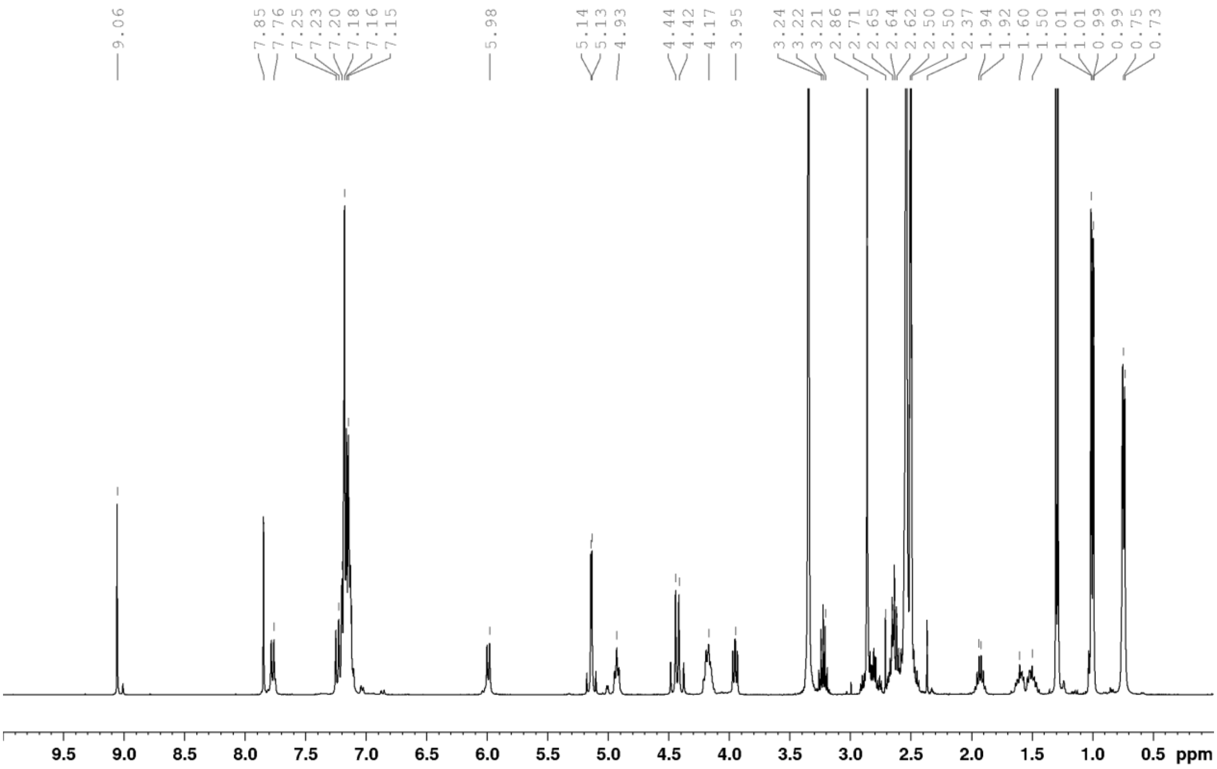

Figure S1: ^{1}H NMR of ritonavir derivative measured in d6-DMSO, ns=32

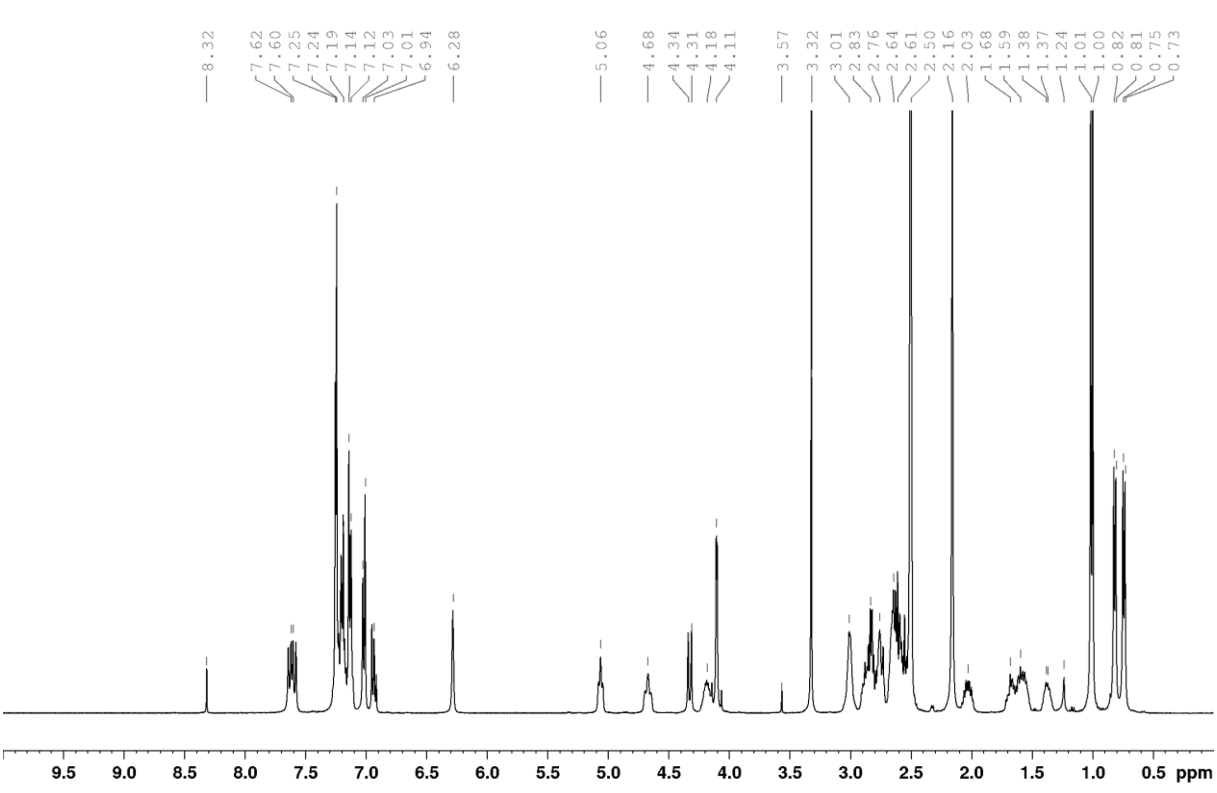

Figure S2: ^{1}H NMR of lopinavir derivative measured in d6-DMSO, ns=32

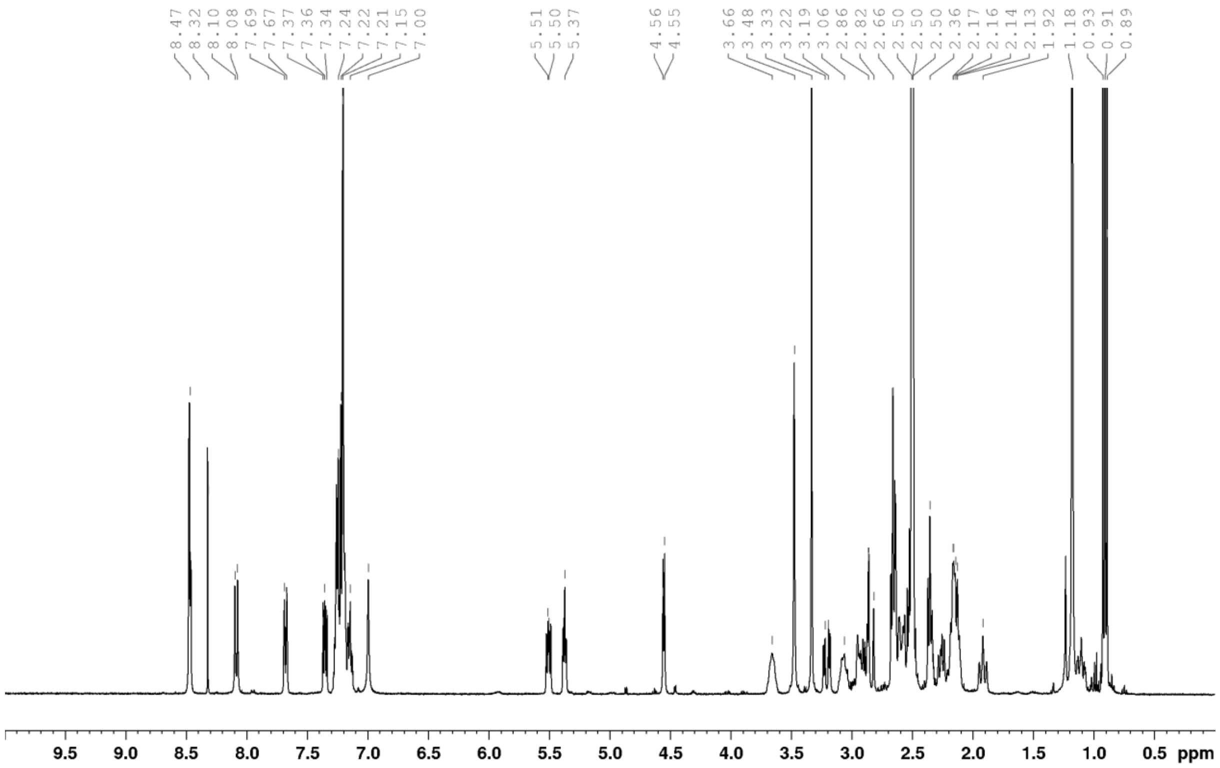

Figure S3: ^{1}H NMR of indinavir derivative measured in d6-DMSO, ns=32

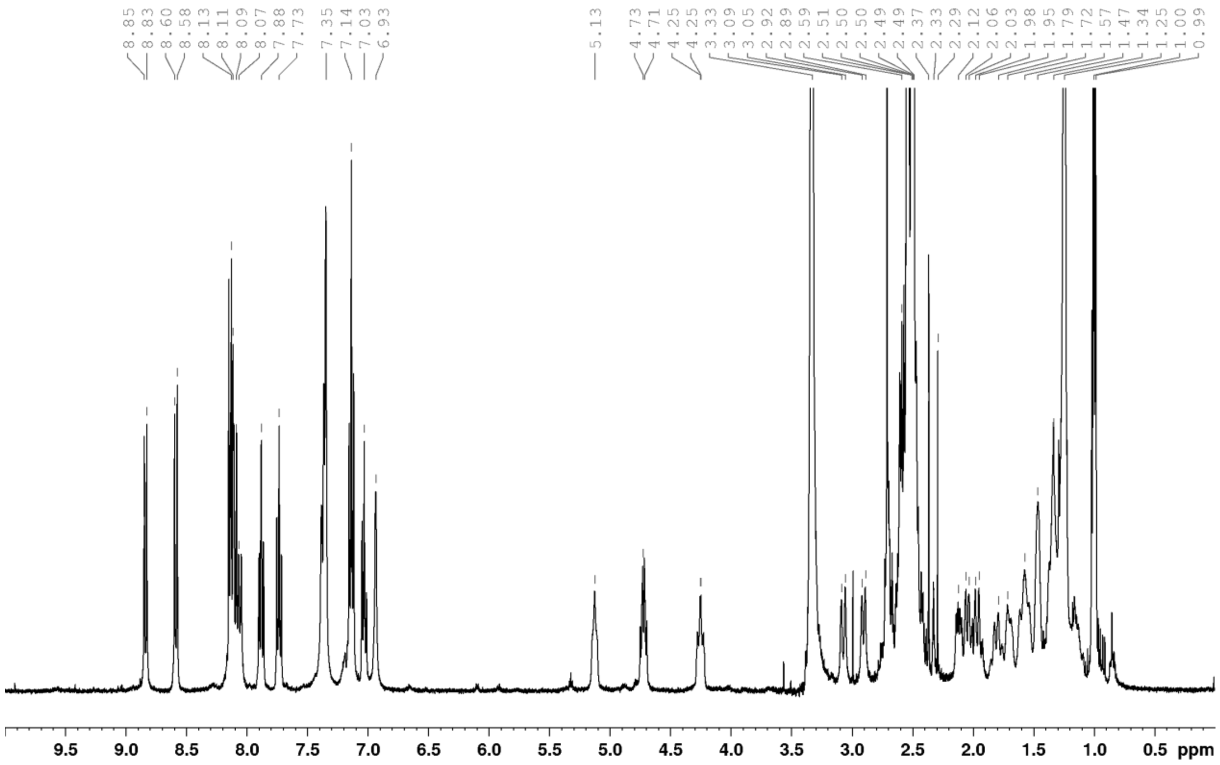

Figure S4: ^{1}H NMR of saquinavir derivative measured in d6-DMSO, ns=32

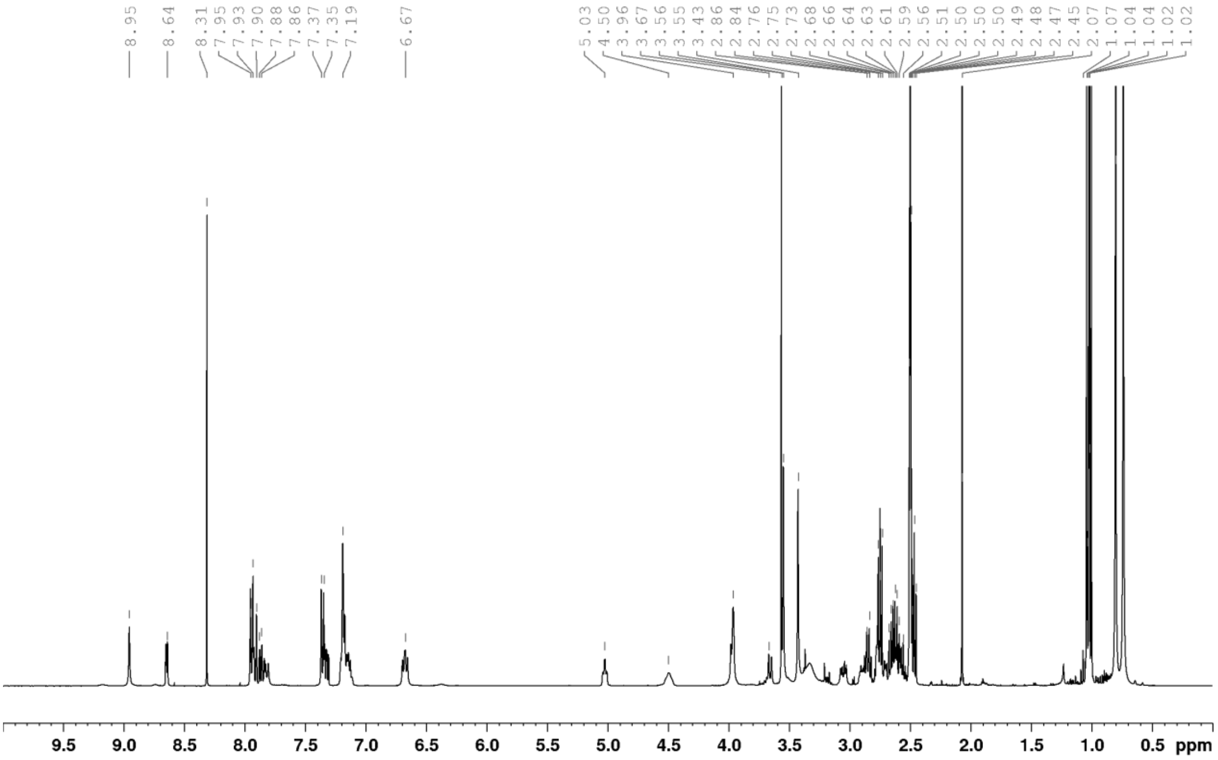

Figure S5: ^{1}H NMR of atazanavir derivative measured in d6-DMSO, ns=32

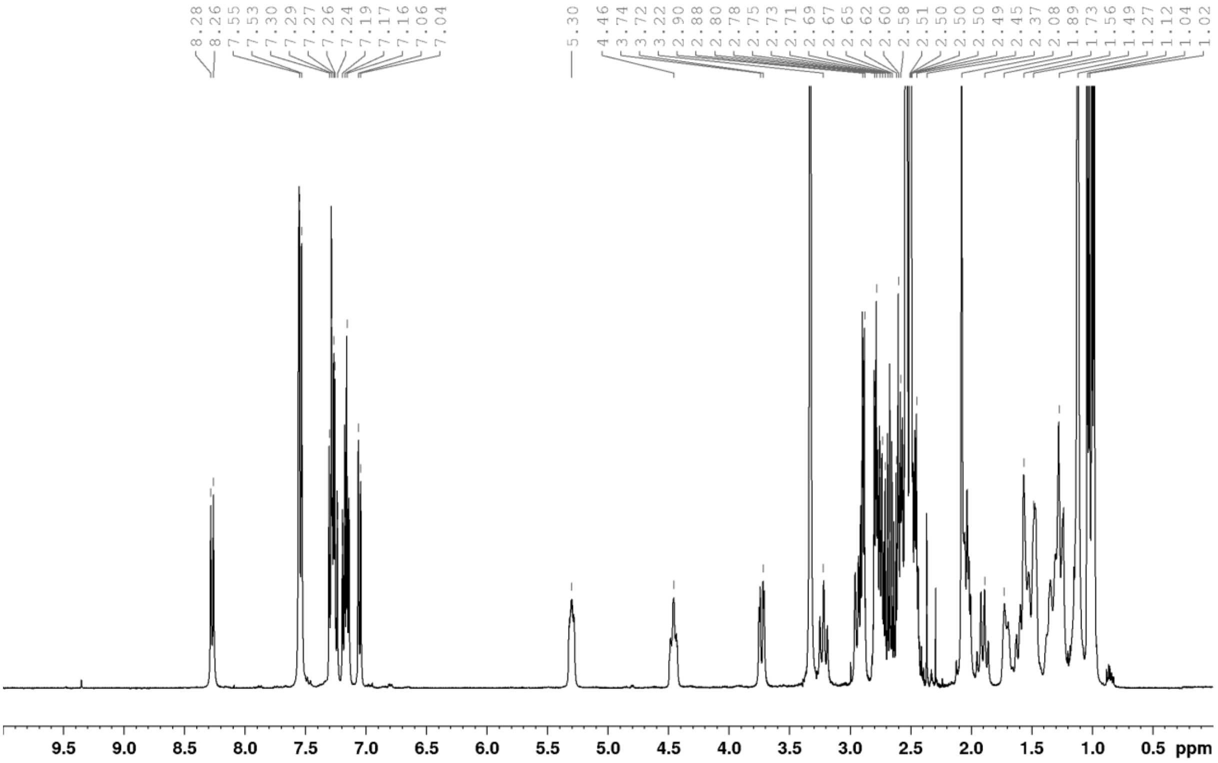

Figure S6: ^{1}H NMR of nelfinavir derivative measured in d6-DMSO, ns=32

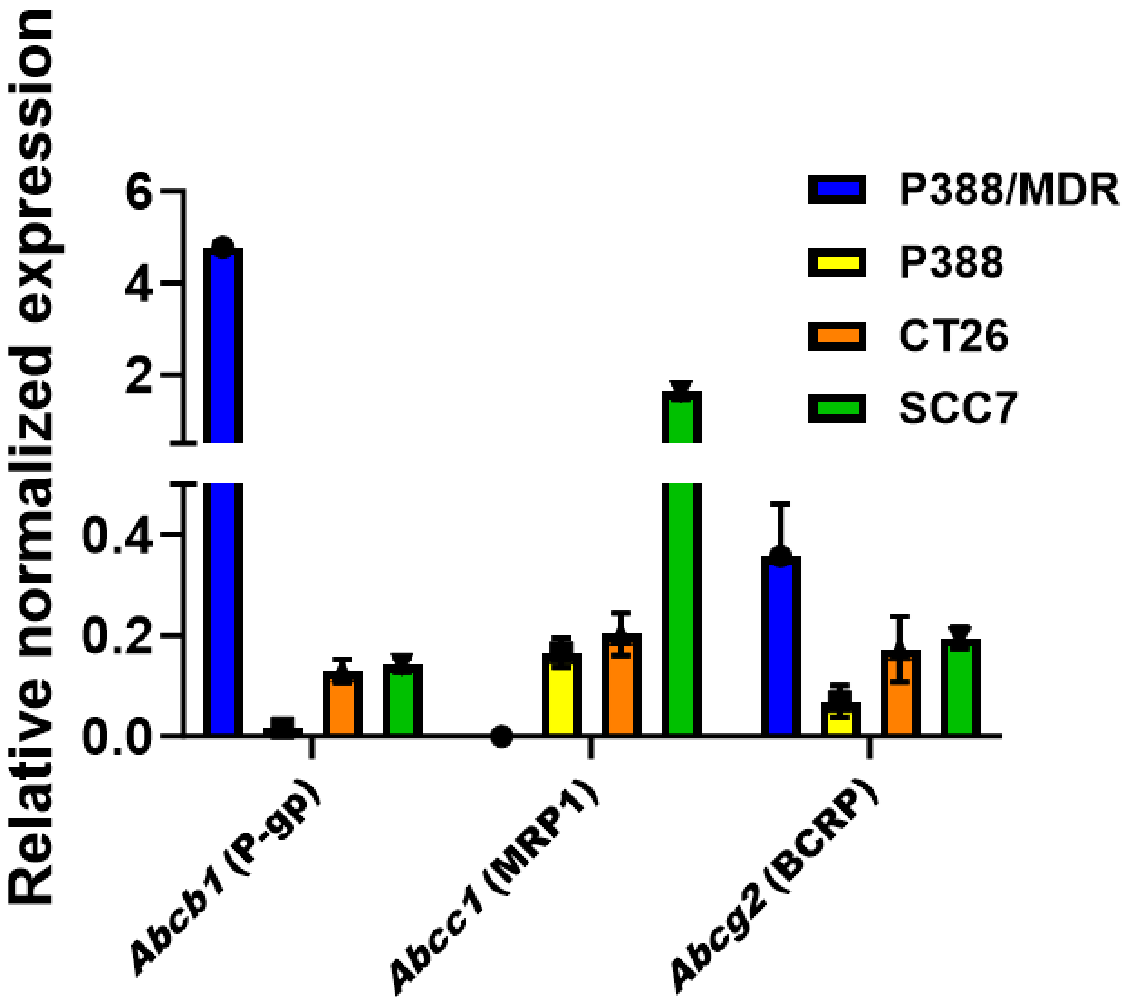

Figure S7. Expression of selected ABC-transporters in cell lines used in the study. Relative normalized expression of genes encoding ABC-transporters *Abcb1* (P-gp), *Abcc1* (MRP1), *Abcg2* (BCRP) in selected murine cancer cell lines determined via RT-qPCR. *ActB*, *CaspC3* and *Hprt* genes were used as internal controls, to which the level of mRNA encoding for tested genes was related during the analysis. Each bar showing relative normalized expression represents mean ± SD of duplicate samples.

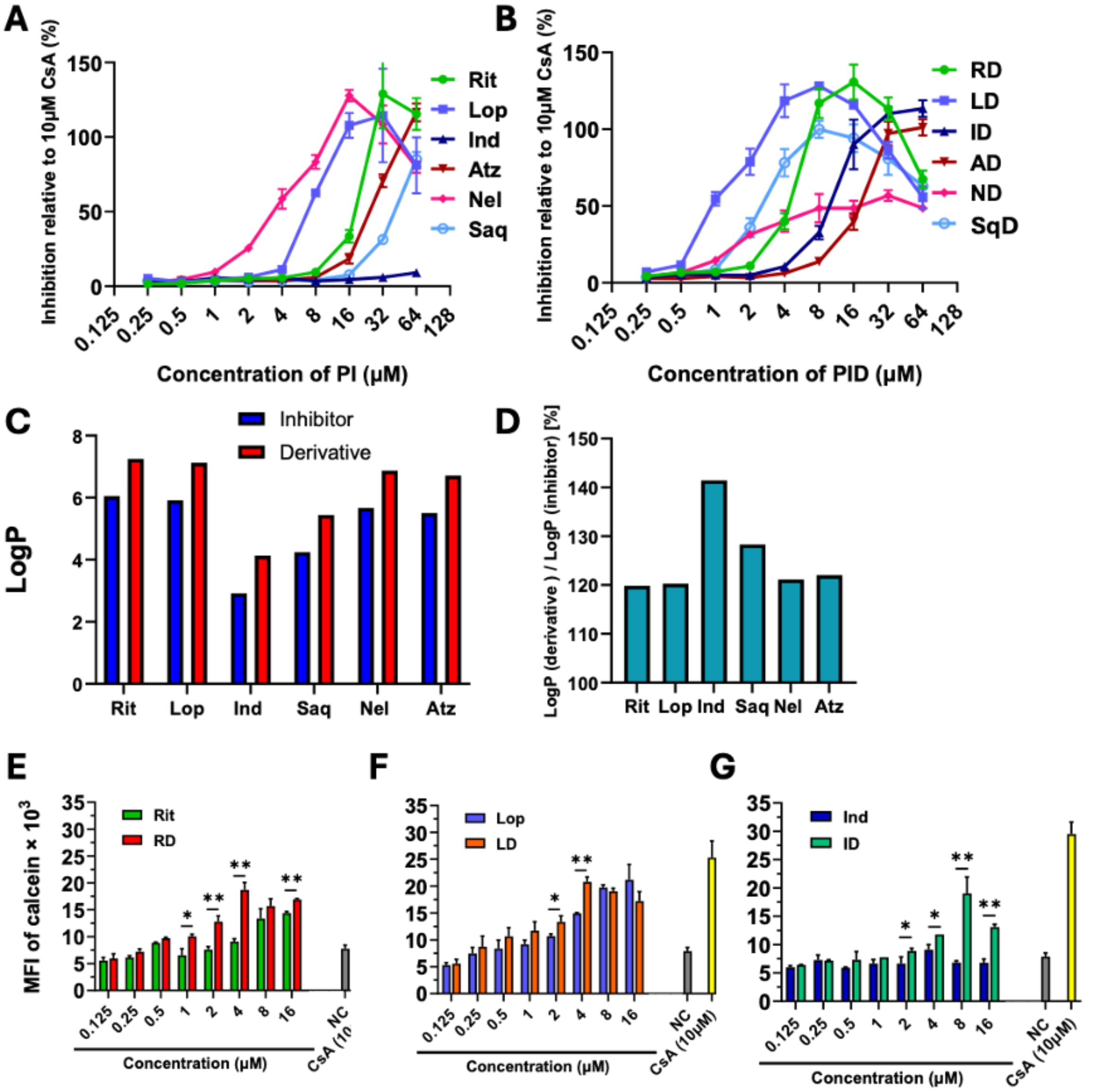

Figure S8. P-gp inhibitory activity and hydrophobicity of selected PIs and PIDs. Inhibition of P-gp activity in P388/MDR cells following 30–min incubation with PIs (A) or PIDs (B) determined using calcein efflux assay and flow cytometry analysis shown as values relative to the P-gp-inhibitory activity of 10 μM cyclosporine A (CsA). Values of octanol/water partition coefficient (LogP) calculated via "XlogP3 Online" software from chemical structures of PIs and PIDs (C) and ratios of LogP for PID and LogP of corresponding PI (D). Inhibition of P-gp activity in CT26 cells after 30 min incubation with Rit and RD (E), Lop and LD (F) or Ind and ID (G) determined using calcein efflux assay and flow cytometry analysis. Cells incubated with

10 μM CsA or only incubation medium were employed as positive control and negative control (NC), respectively. Each bar represents the mean ± standard deviation (SD) of the measured values of mean fluorescence intensity (MFI) of calcein from triplicate samples. Experiments were conducted at least twice and yielded similar results. Statistically significant differences between compared compounds evaluated via unpaired two-tailed Student's *t*-test are indicated by *, **, and ***, denoting $P \leq 0.05$, $P \leq 0.01$, and $P \leq 0.001$, respectively.

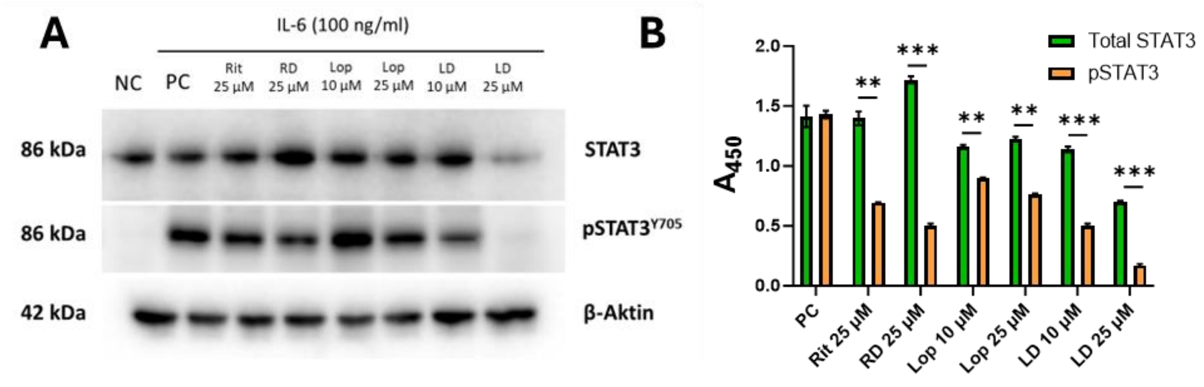

Figure S9. LD potently inhibits STAT3 signaling. Western blot analysis of cell lysates from CT26 cells treated with Rit, RD, Lop and LD (A) with detection of STAT3 and phosphorylated STAT3 (pSTAT3). Cells incubated in culture medium alone were used as negative control (NC), cells treated only with IL-6 were used as positive control (PC), β-actin was used as total protein amount control. ELISA analysis of cell lysates from CT26 cells treated with Rit, RD, Lop and LD (B) with detection of STAT3 and phosphorylated STAT3 (pSTAT3). PC as described above was used. Each bar represents the mean ± standard deviation (SD) of the measured values of the absorbance measured at 450 nm (A_{450}) from triplicate samples. Statistically significant differences between total and phosphorylated STAT3 evaluated via unpaired two-tailed Student's *t*-test are indicated by *, **, and ***, denoting $P \leq 0.05$, $P \leq 0.01$, and $P \leq 0.001$, respectively.

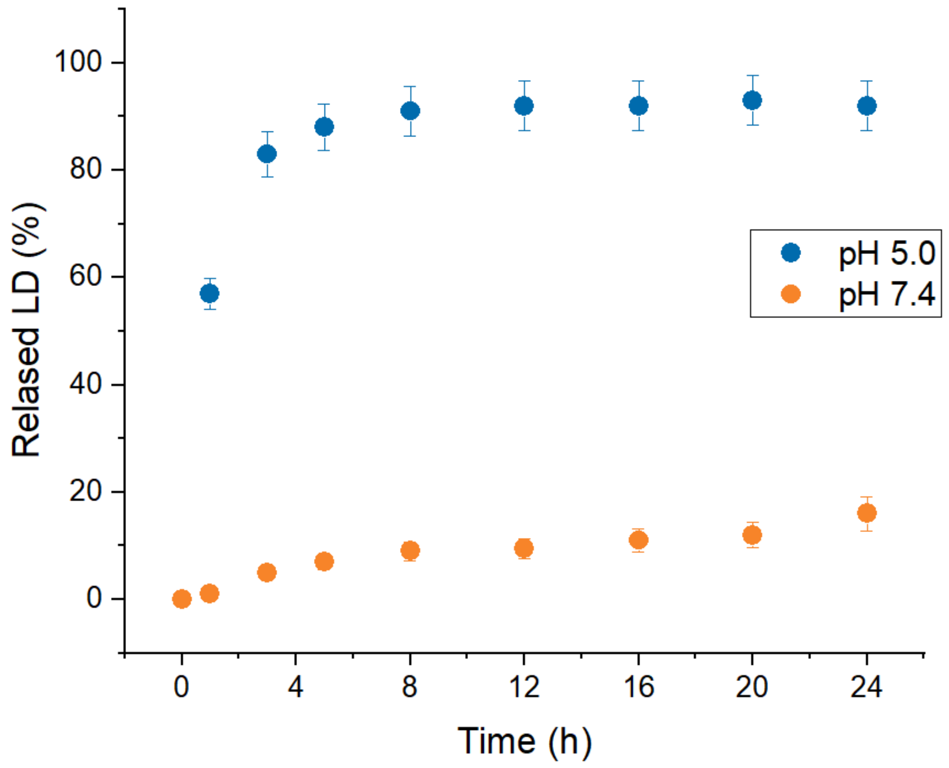

Figure S10. Release profile of LD from HPMA polymer at different pH levels. The graph shows the percentage of LD released over 24 hours at pH 5.0 (blue circles) and pH 7.4 (orange circles). LD release is significantly higher at pH 5.0, indicating acid-facilitated hydrolysis. Data is presented as mean ± SD from triplicate samples.

Table S1. Cytostatic activity of selected PI, PIDs and P-LD in P388/MDR, CT26 and SCC7 cell lines shown as IC_{50} ± SD.

	IC50 (µM ± SD)						
**cell line**	**Lop**	**LD**	**P-LD**	**Rit**	**RD**	**Ind**	**ID**
**P388/MDR**	16.2 ±1.0	10.1 ± 1.1	17.0 ± 5.1	40.8 ± 11.3	15.3 ± 3.7	>128	29.0 ± 12.6
**CT26**	10.2 ± 0.3	4.1 ± 1.4	9.6 ± 0.1	27.9 ± 3.4	12.9 ± 1.4	>128	0.9 ± 0.4
**SCC7**	14.2 ± 0.4	10.9 ± 1.3	11.4 ± 1.1	24.4 ± 1.8	13.2 ± 1.1	>128	1.9 ± 0.6

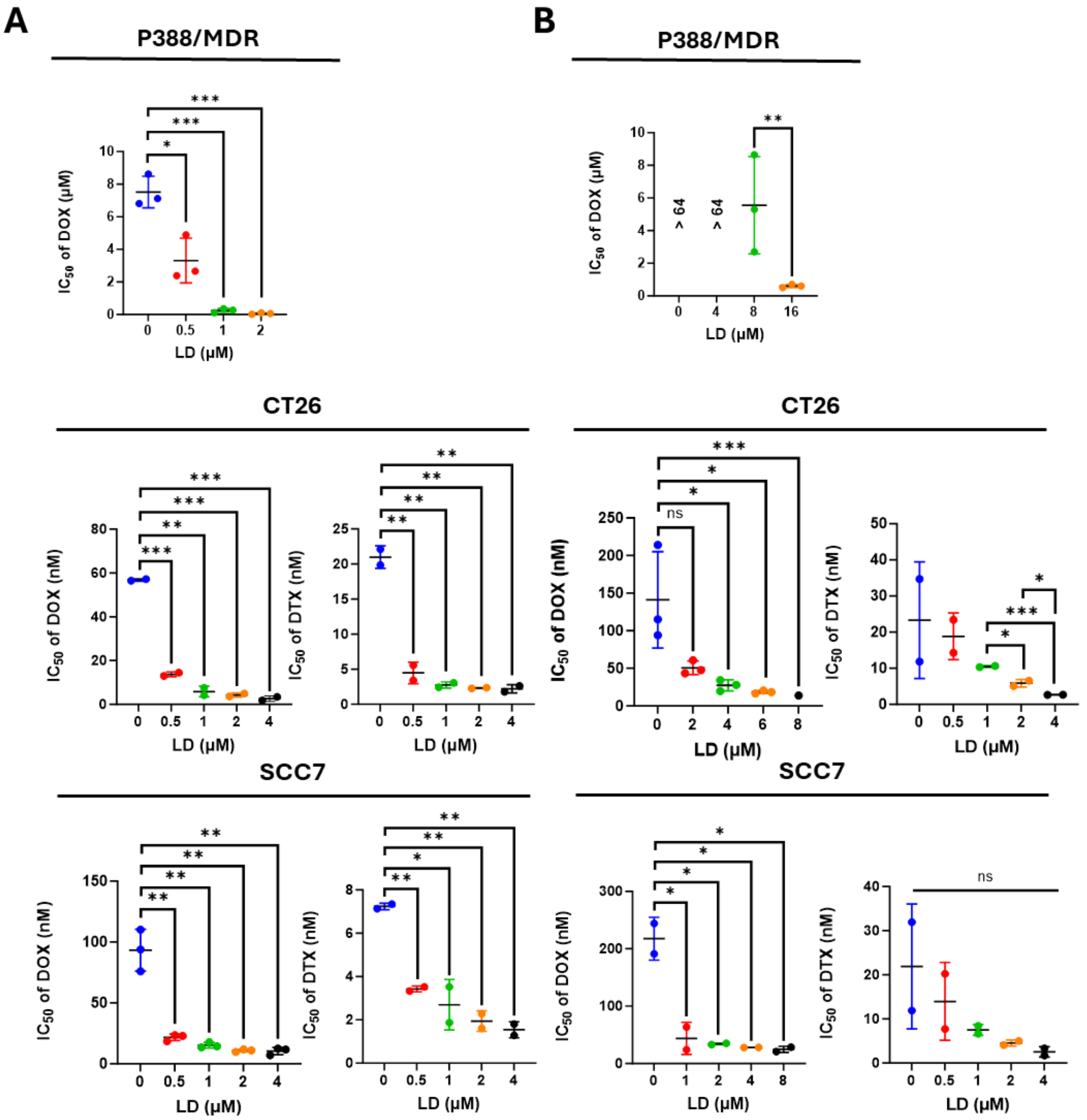

Figure S11. IC_{50} plots corresponding to the curves depicted on Figure 5. IC_{50} determined via [^{3}H]-thymidine incorporation assay after the treatment of cell lines with free cytostatic drugs and free LD (A) or polymeric conjugates (B) and 72 h of incubation, determined via a [^{3}H]-thymidine incorporation assay. Concentrations are shown as free drug equivalents in experiments with polymeric conjugates. Each point represents IC_{50} obtained from a single experiment. SDs for each set of values are shown. Statistically significant differences between compared LD concentrations evaluated via unpaired two-tailed Student's *t*-test are indicated by *, **, and ***, denoting $P \leq 0.05$, $P \leq 0.01$, and $P \leq 0.001$, respectively.

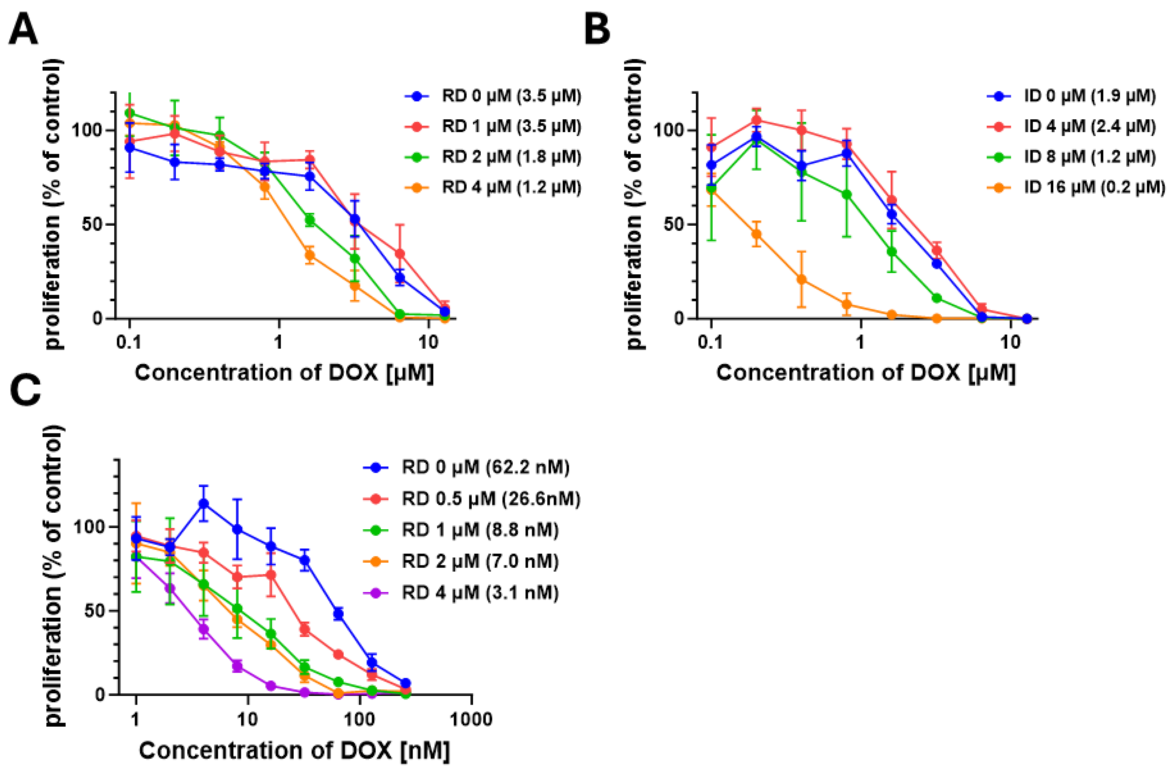

Figure S12. RD and ID possess moderate potential to sensitize P-gp expressing cells to cytostatic activity of DOX. Sensitization of P388/MDR cells to the cytostatic activity of DOX in the presence of RD (A) or ID (B) at various constant concentrations. Sensitization of CT26 cells to the cytostatic activity of DOX with various constant concentrations of RD (C) after 72 h of incubation. [^{3}H]-thymidine incorporation assay was employed herein. Proliferation of cells exposed to the test drugs relative to those exposed to the same concentration of only RD or ID. IC_{50} values for each cytostatic drug in the absence or presence of RD or ID are presented in brackets. Each data point represents the mean ± SD of tetraplicate samples. Each experiment was conducted at least twice, and similar results were obtained.

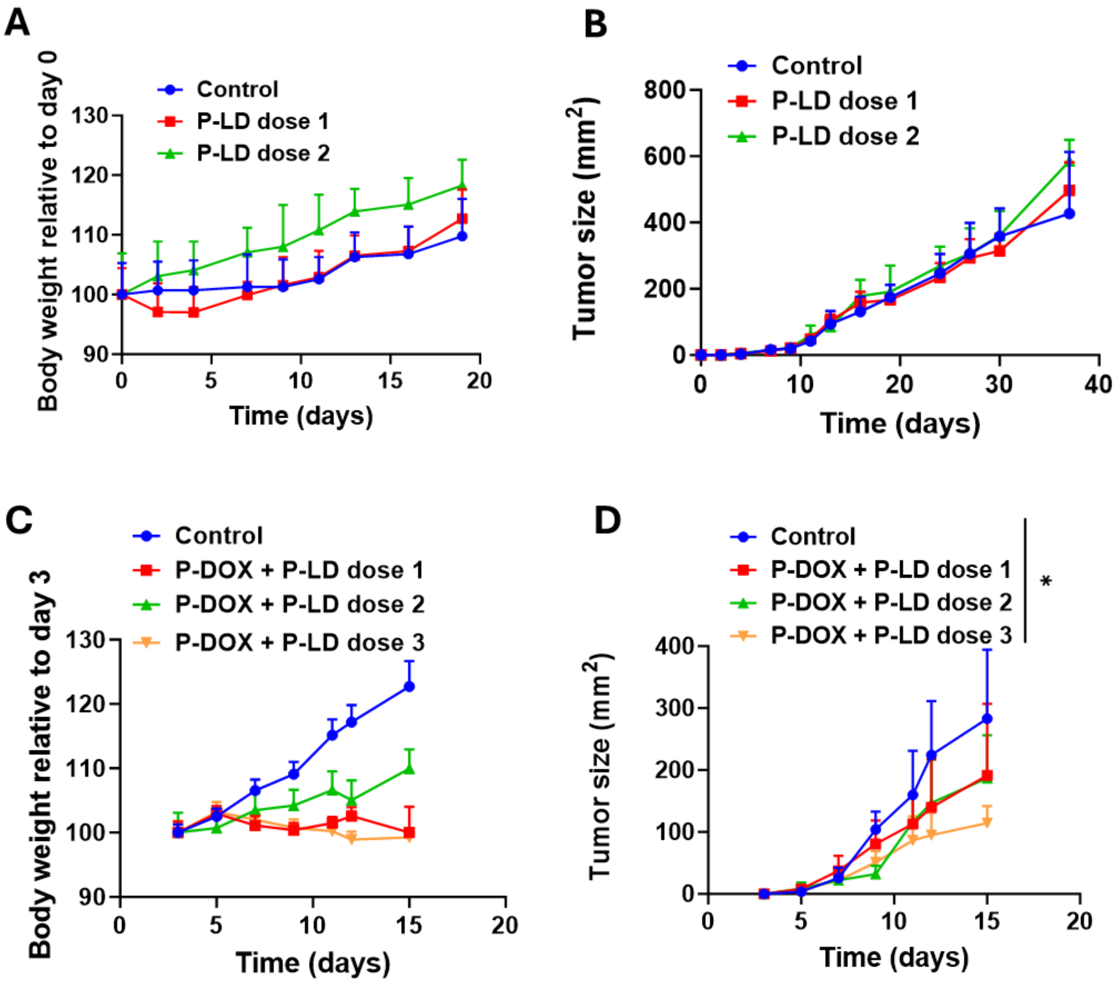

Figure S13. P-LD shows no toxicity and inherent antitumor activity but possesses potential to sensitize highly MDR cancer cells to P-DOX. BALB/c mice (n = 4) were s.c. inoculated with 2.0 × 10^5 CT26 cells on day 0. Mice were i.p. injected with 5 doses of P-LD (150 or 200 mg/kg per dose) on days 8, 10, 12, 14 and 16. Toxicity (A) and tumor growth (B) were monitored in experimental mice during the experiment. Rag2$^{-/-}$ mice (n = 4) were s.c. inoculated with 1 × 10^6 P388/MDR cells on day 0. Mice were i.p. injected with a single dose of P-LD (120, 160 or 200 mg LD/kg) and i.v. injected with single dose of P-DOX (30, 40 or 50 mg DOX/kg). Toxicity (C) and tumor growth (D) were monitored in experimental mice during the experiment. P-DOX was administered 1 h following the administration of P-LD. Mice of the control group were injected with the same volume (250 µl) of phosphate buffered saline. unpaired two-tailed Student's *t*-test and Mantle–Cox log-rank test were employed for analyzing

the statistical significance of the data, which has been indicated by *, **, and *** (denoting $P \leq 0.05$, $P \leq 0.01$, and $P \leq 0.001$, respectively). Experiments were done twice with comparable results.

Table of Contents Graphic

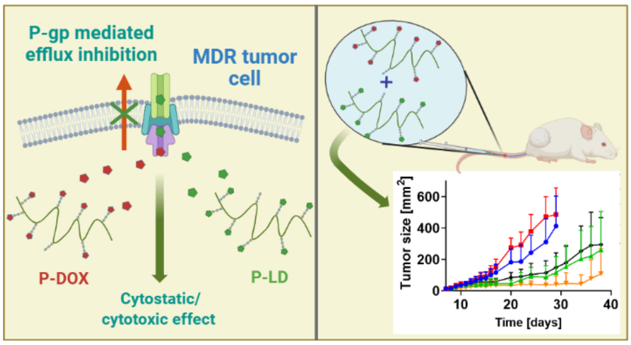

Supplement: Supplementary file 1 [file bm5c02097_si_001.pdf]
